# The social cost of contacts: Theory and evidence for the first wave of the COVID-19 pandemic in Germany

**DOI:** 10.1371/journal.pone.0248288

**Published:** 2021-03-19

**Authors:** Martin F. Quaas, Jasper N. Meya, Hanna Schenk, Björn Bos, Moritz A. Drupp, Till Requate

**Affiliations:** 1 Department of Economics, Leipzig University, Leipzig, Germany; 2 German Centre for Integrative Biodiversity Research (iDiv) Halle-Jena-Leipzig, Leipzig, Germany; 3 Department of Economics, University of Hamburg, Hamburg, Germany; 4 CESifo, Munich, Germany; 5 Department of Economics, Kiel University, Kiel, Germany; Texas A&M University College Station, UNITED STATES

## Abstract

Building on the epidemiological SIR model, we present an economic model with heterogeneous individuals deriving utility from social contacts creating infection risks. Focusing on social distancing of individuals susceptible to an infection we theoretically characterize the gap between private and social cost of contacts. Our main contribution is to quantify this gap by calibrating the model with unique survey data from Germany on social distancing and impure altruism from the beginning of the COVID-19 pandemic. The optimal policy is to drastically reduce contacts at the beginning to almost eradicate the epidemic and keep them at levels that contain the pandemic at a low prevalence level. We find that also in laissez faire, private protection efforts by forward-looking, risk averse individuals would have stabilized the epidemic, but at a much higher prevalence of infection than optimal. Altruistic motives increase individual protection efforts, but a substantial gap to the social optimum remains.

## Introduction

The reduction of physical social contacts (“social distancing”) has been a key measure for public disease control in the COVID-19 pandemic around the world. While social distancing reduces infection rates, it naturally comes at the expense of the lost benefits of contacts. Since the global death toll in the COVID-19 pandemic has been around 5,000 cases a day for most of the year 2020, social distancing is a key factor in containing the virus. We study how contacts should be reduced from the perspective of a social planner and to whether voluntary contact reductions by risk-averse and impure altruistic persons prone to infection would come close to, or substantially differ from, the social optimum.

To address these questions, we extend the SIR (susceptible-infected-recovered) model of epidemiological dynamics [1] by including the behavior of heterogeneous, forward-looking individuals that differ in infection, recovery, and mortality rates (implying heterogeneous baseline reproduction rates), and in their preferences. We keep the analysis simple by focusing on the behavior of susceptible individuals and infected individuals who do not yet know about their infection, considering the behavior of COVID-19 patients (assumed to be strictly quarantined) and recovered individuals as fixed. This focus allows us to contrast a private (‘laissez-faire’) Nash equilibrium with the Pareto-optimal social distancing policy that targets different population groups.

We provide analytical results on the gap between the private and social costs of contacts due to infection externality. We show what drives the gap between purely selfish and socially optimal social distancing and that it decreases with the degree of impure altruism. To quantify the gap between private and socially optimal behavior, we rely on a unique data set from a representative sample of around 3, 500 individuals in Germany at the beginning of the COVID-19 epidemic, and calibrate our model to official epidemiological statistics for Germany.

Our survey elicits reported reductions in physical social contacts and the relative share of impurely altruistic motivation for social distancing, allowing us to derive the social cost of contacts without relying on estimates of the value of a statistical life (VSL) from other contexts, and to separate purely selfish from altruistic motivations. We conducted the survey in late March 2020, when almost all Germans were still susceptible.

Our data collection period includes the introduction of a nationwide ban on contact, which is similar to the “shelter-in-place” policy in the United States. While many social distancing policies aim to reduce mobility, the German contact ban focused specifically on reducing physical contact, leaving considerable scope for voluntary behavior in choosing local contacts in particular. Our survey data is better able to capture such local contact reductions than other data sources such as to mobile phone data, which we also consider for comparison. Furthermore, the timing of our survey allows us to examine private contributions to a public good in the case of social distancing and to test the robustness of the role of regulation. As the severity of COVID-19 differs with age and gender, our application to Germany distinguishes groups along these dimensions.

Our calibrated model provides the following results. First, the optimal social distancing policy drastically reduces contacts to bring infection rates below 1 per 100, 000 at the beginning of the pandemic and stabilizes contacts at about a third of pre-pandemic levels to keep the basic reproduction number stable at one. Second, we find only slight differences in social distancing between groups, both in the laissez-faire equilibrium and in the social optimum.

Third, we find that the social costs of contacts are multiple times the private costs, and the ratio is particularly high at low infection rates. Fourth, we find that impure altruistic behavior fills a substantial part of the gap to the social optimum, with the group-specific reduction of the gap ranging from 28 percent for old men to 32 percent for young women. We also find that altruism has a positive effect on welfare and closes the welfare gap between the laissez-faire equilibrium with selfish individuals and the optimum by about one third. A gap still remains as the motivation to protect oneself continues to be the main determinant of individual actions in spite of some altruistic motives coming into play.

Finally, we show that purely selfish protection reduces the number of contacts to a level that keeps the basic reproduction number at one, albeit at a prevalence of the disease that is much higher than optimal. Accordingly, the death toll in the laissez-faire Nash equilibrium is about 20 times higher than in the social optimum. These findings are in line with general theory according to which self-protection by risk-averse individuals can contribute to alleviating the problem of external effects in a setting characterized by substantial private risk [2].

Whereas the literature is rapidly expaning, to the best of our knowledge, our paper is the first to (i) combine a heterogeneous, group-specific analytical model with survey data on individual behavioural change to quantify the gap between the social optimum and the Nash equilibrium with risk-averse, selfish individuals (‘laissez-faire’); (ii) estimate welfare effects based on empirical evidence, while disentangling purely selfish and altruistic components of social-distancing behavior. While so far most economic-epidemiological models are calibrated to US data, our application to Germany offers an interesting complementary case study, as an advanced economy that has managed the first month of the pandemic with relatively few deaths and relatively modest regulations.

## Related literature and contribution

Our research adds to the rapidly growing literature on the economics of epidemics applied to the ongoing COVID-19 pandemic. It draws on earlier contributions on the economics of infectious diseases [3–11]. In particular, we build on Fenichel et al. [5] regarding socially optimal and impure altruistic behavior with heterogeneous groups. Our theoretical analysis, comparing the Nash equilibrium dynamics with individual decentralized decisions and the social planner’s solution, is similar to the approach of Fenichel [6].

Our contribution regarding the stylized purely selfish and impure altruistic private versus social cost of contacts with heterogeneous groups is most closely related to recent work by Farboodi et al. [12] and Acemoglu et al. [13]. Faarbodi et al. [12] study an optimal control model with a single type of agent to compare contacts in a laissez-faire equilibrium to a social planner’s solution fully internalizing the externality. The authors also compare pure selfish behavior with imperfect altruism. They calibrate their model based on the literature, including VSL estimates from [14], finding that a laissez-faire equilibrium comes close to the decline in social activity as measured in US micro-data from SafeGraph. Their optimal policy, which accounts for the infection externality, would stabilize contacts at about 60 percent of pre-pandemic levels. In comparison to our work, they do not disentangle selfish and altruistic behavior and capture group heterogeneities. With a similar focus, Bethune et al. [15] study the infection externalities and compare individual behavior with the social optimum in a SIR model calibrated using VSL estimates. For the US, they estimate the social cost of infections to be 3.5 fold higher than the private cost. They find that, in contrast to the laissez-faire equilibrium, the social planner would eradicate the disease, except if it’s social cost is very small. Eichenbaum et al. [16] use the SIR model in a representative agent setting to show that the equilibrium of selfish individuals is not Pareto efficient, as individuals take infection rates as given.

Acemoglu et al. [13] extend the SIR model to heterogeneous groups and provide a closed-form solution of the dynamic model. Specifically, their ‘Multi-Risk’ model considers different age classes that differ in their infection, hospitalization and mortality rates. In their calibration for the US, they specify parameters based on the literature and account for heterogeneity in some parameters across age groups, distinguishing young (20–44), middle-aged (45–65) and old (> 65). They find that a targeted, group-specific social distancing policy reduces economic cost and lives lost compared to an undifferentiated policy. Building on this Multi-Risk SIR model, Gollier [17] compares welfare effects of a ‘suppression’ policy where the disease is eradicated, with a ‘flatten the curve’ policy, where infections are only kept below the capacities of the health systems. The model is calibrated for France, considering three age groups: young (0-18), middle-aged (19-64), and old (> 65). Gerlagh [18] considers heterogeneity in preferences about social contacts, health cost or transmission rates in a simplified SIR model. He shows that a group-specific optimal social distancing policy sets tighter distancing policies for elderly when based on health characteristics, but sets tighter distancing policies for the young when based on the transmission of the virus. Overall, he finds that public benefits of optimal social distancing are an order of magnitude higher than the private benefits. Grimm et al. [19] extend the SEIR model for, among others, heterogeneous infectiousness parameters and solve it numerically with calibration from the literature for Germany.

Several other recent papers extend the SIR model to study social distancing behaviour and optimal policy response in the COVID-19 pandemic with different foci [20–27]. Of these, Alfaro et al. [28] is most closely related to our paper. They use a homogenous SIR model to show that infected individuals internalise part of the infection externality due to altruistic preferences. Yet, their data does not allow for clearly disentangling to what extent altruistic motives narrow the gap between selfish and socially optimal behavior. There is also a group of papers studying macroeconomic effects, such as fiscal consequences or income shocks related to the effects on trade or supply chains [29–33]. In relation to income losses, which our surveyed households expect on average, our empirical strategy assumes that—as far as income depends on physical contacts—these income losses are captured by their individual reductions in contacts.

Finally, our paper relates to the literature on the private provision of a public good under uncertainty [2, 34–37] and public good provision under impure altruism [38–41], as it provides evidence for a general hypothesis that uncertainty can help mitigate the externalities problem.

## Economic-epidemiological model with heterogeneous groups

### Epidemiological dynamics

We draw on the canonical epidemiological SIR model [1], augmented by additional equations to include quarantine, and set up in discrete time. Total population in period *t*, denoted by *N*_*t*_, splits up into susceptibles, *S*_*t*_, infected and infectious, who do not yet have any symptoms and do not know they are infected, *I*_*t*_, COVID-19 patients who are in quarantine, *Q*_*t*_, and recovereds, *R*_*t*_. We also record the number of deads *D*_*t*_, such that *N*_*t*_ = *S*_*t*_ + *I*_*t*_ + *Q*_*t*_ + *R*_*t*_ = *N*_0_ − (*D*_*t*_ − *D*_0_). Recovereds are assumed to be immune.

We model heterogeneous population groups *j* that differ in socio-demographic characteristics, notably age and gender, risk exposure, and preferences. Considering this heterogeneity addresses limitations of the aggregate SIR model [42], and allows studying how incentives to choose frequencies of contacts with others *c*_*jt*_ differ with these characteristics. Different frequencies of contacts result in heterogeneous effective infection rates. Individuals from different groups may also differ in their clinical course of the infection, resulting in heterogeneous fatality or recovery rates. The current state of the epidemic is determined by the number of susceptibles *S*_*jt*_, infected *I*_*jt*_, quarantined *Q*_*jt*_, and recovered *R*_*jt*_ from all groups *j*. We use the symbols without group index to denote aggregate values, i.e. *I*_*t*_ ≔ ∑_*j*_ *I*_*jt*_ is the aggregate total number of infected, and so on. To keep the model tractable, we assume that individuals are homogeneous within a group and do not switch groups. The epidemiological dynamics how individuals of all groups change their health status are described by:

$$\begin{array}{cc} S_{j,t+1} & =S_{jt}-\beta \left(c_{jt}\right)\;S_{jt}\;I_{t}, \end{array}$$ (1a)

$$\begin{array}{cc} I_{j,t+1} & =\left(1-\theta_{j}-\alpha_{j}^{i}-\gamma_{j}^{i}\right)\;I_{jt}+\beta \left(c_{jt}\right)\;S_{jt}\;I_{t}, \end{array}$$ (1b)

$$\begin{array}{cc} Q_{j,t+1} & =\left(1-\alpha_{j}^{q}-\gamma_{j}^{q}\right)\;Q_{jt}+\theta_{j}\;I_{jt}, \end{array}$$ (1c)

$$\begin{array}{cc} R_{j,t+1} & =R_{jt}+\gamma_{j}^{i}\;I_{jt}+\gamma_{j}^{q}\;Q_{jt}, \end{array}$$ (1d)

where *β*(*c*_*jt*_) is the infection rate given the frequency of physical social contacts *c*_*jt*_ of susceptibles of group *j*. In general this term represents a matching function which could depend on the activities of those searching for contacts and those being available to be contacted. Here we assume that only the individual searching contacts affect the probability of an infection, thus *β*(*c*_*jt*_) depends on *c*_*jt*_ only. Our quantitative results are robust to alternative specifications of a matching function that is homogeneous of degree one, the standard assumption in economic matching models [43]. The given assumption implies that the health externality is fully captured by the difference in the individual and social value of an infection, see section.

We further specify *β*(*c*_*jt*_) = *βc*_*jt*_, which means that the probability of getting infected is proportional to the number of physical social contacts. We assume that the infected who do not yet know about the infection behave just like susceptibles. With rate *θ*_*j*_, an infection becomes evident, as the COVID-19 patient shows symptoms, or as a test turned out to be positive. We assume that once the infection is detected, the COVID-19 patient goes into strict quarantine and does not infect others any more. In principle, *θ*_*j*_ is a policy variable as well, as this parameter can be influenced by testing frequencies, among others. Given our focus on social distancing, we consider *θ*_*j*_ as exogeneously given in this paper. Moreover, $\gamma_{j}^{i}$ and $\gamma_{j}^{q}$ are the recovery rates, while $\alpha_{j}^{i}$ and $\alpha_{j}^{q}$ are the COVID-19 mortality rates, of infected and quarantined individuals from group *j* die. While it is straightforward to include non COVID-19-related mortality in the model, we ignore it here.

The group-specific basic reproduction numbers, i.e. the number of people infected by one individual from group *j* on average, are $R_{j0}=\beta \;c_{j0}/\left(\alpha_{j}^{i}+\gamma_{j}^{i}+\theta_{j}\right)$. The overall basic reproduction number $R_{0}$, is the mean of group specific basic reproduction numbers, weighted by the initial fraction of susceptibles from the respective groups, $R_{0}=\sum_{j}R_{j0}\;S_{j0}/N_{j0}$. Note that the basic reproduction number $R_{0}$ is a function of contacts and can thus be reduced by voluntary or mandatory social distancing.

### Nash equilibrium dynamics with private self-protection

A key interest of our paper is in the choice of physical social contacts by susceptibles who we model as forward-looking expected utility maximizers. For both individuals and society we assume a finite planning horizon of *T* weeks. After *T* weeks, group *j* individuals incur the present value utility level $V_{j}^{n}$, with superscript *n* denoting the no-epidemic situation. Our focus is on a first ‘wave’ of the pandemic. Thus we keep epidemiological and economic parameters constant in the model, and consider *T* to be sufficiently long such that the finite time horizon does not have a direct effect on the dynamics during that first wave.

Following [5] and [6], each individual takes as given the time paths of *S*_*jt*_, *I*_*jt*_, *Q*_*jt*_ and *R*_*jt*_, for all groups *j*. We use $V_{jt}^{h}$ to denote the value function for an individual of group *j* in health state *h* ∈ {*s*, *i*, *q*, *r*, *d*} at time *t*, i.e. the expected present value of utility the individual attaches to reaching health state *h*. As usual, the model is solved backwards, starting with the final potential health states, recovered or dead.

The value function in the recovered health state *r* is given by

$$\begin{array}{c} V_{jt}^{r}=u_{j}^{r}+\delta_{j}\;V_{j,t+1}^{r}, \end{array}$$ (2)

where *δ*_*j*_ ∈ (0, 1) denotes the utility discount factor, $u_{j}^{r}$ the Bernoulli utility of recovereds. It follows that the value of being recovered $V_{j}^{r}$ is independent of the state of the epidemic and equal to

$$\begin{array}{c} V_{jt}^{r}=\frac{u_{j}^{r}}{1-\delta_{j}}\;(1-\delta_{j}^{T-t})+V_{j}^{n}\;\delta_{j}^{T-t}, \end{array}$$ (3)

which is a weighted average between the infinite time-horizon value function for recovereds, the first fraction on the right-hand-side of (3), and the value of an individual in the no-epidemic situation, $V_{j}^{n}$. The weighting factor on the first component decreases, and the weighing factor on the second component increases, as the arrival time *T* of the vaccination approaches.

An infected individual from group *j* will recover with probability $\gamma_{j}^{q}$ and die with probability $\alpha_{j}^{q}$, both of which are, by assumption, independent of the state of the epidemic, but vary with individual characteristics, such as age and general health conditions. In the following we use $u_{j}^{i}$ to denote a group *j* individual’s Bernoulli utility function in health state *i*. The corresponding value function is determined by

$$\begin{array}{c} V_{jt}^{q}=u_{j}^{q}+\delta_{j}\;\{(1-\gamma_{j}^{q}-\alpha_{j}^{q})\;V_{j,t+1}^{q}+\gamma_{j}^{q}\;V_{jt}^{r}+\alpha_{j}^{q}\;V_{j}^{d}\}, \end{array}$$ (4)

which is the sum of the utility of being in quarantine plus the discounted expected utility of staying in quarantine, recovering, or dying, using $V_{j}^{d}$ to denote the present (dis-)utility value of death. The term in curly brackets is the (von Neumann–Morgenstern) expected utility of either remaining in quarantine, recovering, or dying. Also $V_{jt}^{q}$ is independent of the state of the epidemic in terms of the number of susceptible, infected, or recovered individuals. Solving (4), we obtain:

$$\begin{array}{c} V_{jt}^{q}=\frac{u_{j}^{q}+\delta_{j}\;\gamma_{j}^{q}\;V_{jt}^{r}+\delta_{j}\;\alpha_{j}^{q}\;V_{j}^{d}}{1-\delta_{j}\;(1-\gamma_{j}^{q}-\alpha_{j}^{q})}\;(1-{\left(\delta_{j}\;\left(1-\alpha_{j}^{q}-\gamma_{j}^{q}\right)\right)}^{T-t})+V_{j}^{n}\;{\left(\delta_{j}\;\left(1-\alpha_{j}^{q}-\gamma_{j}^{q}\right)\right)}^{T-t}. \end{array}$$ (5)

Note that $V_{jt}^{q}$ can be interpreted in terms of quality-adjusted life years. It is increasing in the quality of life, as measured by the utility levels $u_{j}^{q}$ and $u_{j}^{r}$. Moreover, $V_{jt}^{q}$ is monotonically decreasing with the ‘severity’ of the disease. More precisely, the value an individual attaches to an infection is monotonically decreasing in the COVID-19 mortality rate $\alpha_{j}^{q}$. This is shown by differentiating (5) with respect to $\alpha_{j}^{q}$, and using that individuals prefer to be infected over being dead, expressed in momentary utility as $u_{i}>\left(1-\delta_{j}\right)\;V_{j}^{d}$. They prefer to be recovered over being dead, expressed in present values as $V_{jt}^{r}>V_{j}^{d}$, and they prefer to be in the situation with no epidemic compared to being infected.

The value an individual attaches to an infection is also monotonically increasing in the Bernoulli utility in the infected state, $u_{j}^{q}$. Differences in the Bernoulli utility functions, a susceptible individual attaches to the different health states, capture the effect of risk aversion. The more averse against health risk an individual is, the smaller will be $u_{j}^{q}$ relative to the utility in the susceptible health state. Thus, the expected present value an individual attaches to an infection is decreasing with the individual’s (health-related) risk aversion. The value function for the unknowingly infected is

$$\begin{array}{c} V_{jt}^{i}=u_{j}^{s}\left(c_{jt}\right)+\delta_{j}\;\{\left(1-\theta_{j}-\gamma_{j}^{i}-\alpha_{j}^{i}\right)\;V_{j,t+1}^{i}+\theta_{j}\;V_{j,t+1}^{q}+\gamma_{j}^{i}\;V_{jt}^{r}+\alpha_{j}^{i}\;V_{j}^{d}\}, \end{array}$$ (6)

where *c*_*jt*_ is the same choice of contacts as the susceptible, as an individual does not know about a potential infection. We now turn to this choice of physical social contacts.

Recall that *βc*_*jt*_ *I*_*t*_ is the rate at which susceptibles get infected after having had physical contacts with infected. This infection rate increases with the frequency *c*_*jt*_ with which susceptible individuals search for physical contacts with others, i.e. *β* > 0. The value function for the individual in state *s* is determined by the Bellman equation:

$$\begin{array}{c} V_{jt}^{s}=\max_{\left\{c_{jt}\right\}}[u_{j}^{s}\left(c_{jt}\right)+\delta_{j}\;\{(1-\beta \;c_{jt}\;I_{t})\;V_{j,t+1}^{s}+\beta \;c_{jt}\;I_{t}\;V_{j,t+1}^{i}\}], \end{array}$$ (7)

with $V_{jt}^{i}$ given in (6) and where utility $u_{j}^{s}$ in state *s* is a concave function of contacts *c*_*jt*_.

In the absence of regulation, and given *S*_*jt*_, *I*_*jt*_, *Q*_*jt*_, and *R*_*jt*_, for all groups *j* and at each point in time, a purely selfish individual chooses the frequency of physical social contacts, *c*_*jt*_, to solve (7). The corresponding first-order condition is given by

$$\begin{array}{c} {u_{j}^{s}}^{\prime }\left(c_{jt}\right)=\delta_{j}\;\beta \;I_{t}\;\{V_{j,t+1}^{s}-V_{j,t+1}^{i}\}. \end{array}$$ (8)

An individual reduces physical social contacts such that her private marginal costs (lost marginal utility of *c*_*jt*_) equals the expected marginal benefit in terms of extending the time enjoying utility $V_{jt}^{s}$ rather than $V_{jt}^{i}$, the expected present value of an infection. The marginal benefit of reducing contacts is the discounted additional utility of staying susceptible weighted by the decreased rate of getting infected, *βI*_*t*_, due to reductions in contacts *c*_*jt*_. If utility is concave in contacts, i.e. ${u_{j}^{s}}^{\prime \prime }\left(c_{jt}\right)<0$, a decrease of ${u_{j}^{s}}^{\prime }\left(c_{jt}\right)$ corresponds to an increase in *c*_*jt*_. It directly follows from (8) that physical social contacts of susceptible individuals decrease with the current number of infected in the population *I*_*t*_. Moreover, contacts of susceptible individuals *c*_*jt*_ decrease with the difference of an individual’s expected present value utility of staying susceptible rather than becoming infected, i.e. $V_{j,t+1}^{s}-V_{j,t+1}^{i}$.

According to (8), the individually optimal contacts, *c*_*jt*_, depend on the number of infected in the entire population, *I*_*t*_. We consider the dynamics of *c*_*jt*_ in open-loop Nash equilibrium, where all individuals take as given the epidemiological dynamics, resulting from the behavior of all others.

### Utilitarian optimum

The social objective we consider is to maximize the sum of expected present values of individual utilities over the frequency of contacts of all individuals and at all time periods, i.e. the utilitarian welfare function. The function is based on the aggregation of unit comparable individual utility functions [44]. To construct unit comparable utility functions for the individuals, we normalize individual utility functions such that momentary utility prior to the COVID-19 pandemic is identical for all individuals, i.e. ${\text{max}}_{c_{j}}u_{j}^{s}\left(c_{j}\right)=\max_{c_{l}}u_{l}^{s}\left(c_{l}\right)$ for all *j*, *l*. Given unit comparability, the utilitarian welfare function is a particularly appealing specification, as it is consistent with the assumptions that social preferences satisfy the von Neumann Morgenstern axioms and the Strong Pareto assumption, i.e., society prefers one allocation over another one if all individuals weakly prefer it and at least one individual strictly prefers it [45]. To take into account that COVID-19 is a potentially deadly disease, we also include an annuity $u_{j}^{d}$ on the present value (dis-utility) an individual attaches to dying, $u_{j}^{d}:=\rho_{j}\;V_{j}^{d}$, where *ρ*_*j*_ is the discount rate corresponding to goup *j*′s discount factor *δ*_*j*_. As before, and in line with the standard approach in social welfare functions [44], we only consider the purely selfish part of individual utility:

$$\begin{array}{c} \hat{W}=\max_{\left\{c_{jt}\right\}}\sum_{j}\sum_{t=0}^{T}\delta_{j}^{t}\;(\left(S_{jt}+I_{jt}\right)\;u_{j}^{s}\left(c_{jt}\right)+Q_{jt}\;u_{j}^{q}+R_{jt}\;u_{j}^{r}+D_{jt}\;u_{j}^{d}), \end{array}$$ (9)

subject to the epidemiological dynamics given by (1). Whereas each individual faces risks of changing their health status, at the societal level the epidemiological dynamics are deterministic. Thus, the problem (9) to find the utilitarian optimum is a standard deterministic dynamic optimization problem that can be solved by the Lagrangian method, using $\lambda_{jt}^{h}$ as the Lagrangian multiplier for the number of individuals in health state *h* ∈ {*s*, *i*, *q*, *r*, *d*} in period *t* + 1. These Lagrangian multipliers have the interpretation of the social value, in units of utility, of an extra individual from group *j* in health state *h*. They are the social equivalent to the value $V_{j,t+1}^{h}$ an individual attaches to the health state *h* in period *t* + 1. The conditions characterizing the socially optimal physical social contacts $c_{jt}^{⋆}$ under epidemiological dynamics can be written as (see Appendix in S1 File)

$$\begin{array}{cc} {u_{j}^{s}}^{\prime }\left(c_{jt}^{⋆}\right)+\delta_{j}\;\frac{S_{jt}}{S_{jt}+I_{jt}}\;\beta \;I_{t}\;(\lambda_{jt}^{i}-\lambda_{jt}^{s}) & =0 \end{array}$$ (10a)

$$\begin{array}{cc} u_{j}^{s}\left(c_{jt}^{⋆}\right)-\lambda_{j,t-1}^{s}+\delta_{j}\;(1-\beta \;c_{jt}^{⋆}\;I_{t})\;\lambda_{jt}^{s}+\delta_{j}\;\beta \;c_{jt}^{⋆}\;I_{t}\;\lambda_{jt}^{i} & =0 \end{array}$$ (10b)

$$\begin{array}{cc} u_{j}^{s}\left(c_{jt}^{⋆}\right)-\lambda_{j,t-1}^{i}+\delta_{j}\;(\left(1-\theta_{j}-\gamma_{j}^{i}-\alpha_{j}^{i}\right)\;\lambda_{jt}^{i}+\theta_{j}\;\lambda_{jt}^{q}+\gamma_{j}^{i}\;\lambda_{jt}^{r}+\alpha_{j}^{i}\;\lambda_{jt}^{d}) & =\sum_{l}\;\delta_{l}\;\beta \;c_{lt}^{⋆}\;S_{lt}\;(\lambda_{lt}^{s}-\lambda_{lt}^{i}) \end{array}$$ (10c)

$$\begin{array}{cc} u_{j}^{q}-\lambda_{j,t-1}^{q}+\delta_{j}\;\left(1-\gamma_{j}^{q}-\alpha_{j}^{q}\right)\;\lambda_{jt}^{q}+\delta_{j}\;\gamma_{j}^{q}\;\lambda_{jt}^{r}+\delta_{j}\;\alpha_{j}^{q}\;\lambda_{jt}^{d} & =0 \end{array}$$ (10d)

$$\begin{array}{cc} u_{j}^{r}-\lambda_{j,t-1}^{r}+\delta_{j}\;\lambda_{jt}^{r} & =0 \end{array}$$ (10e)

$$\begin{array}{cc} u_{j}^{d}-\lambda_{j,t-1}^{d}+\delta_{j}\;\lambda_{jt}^{d} & =0, \end{array}$$ (10f)

with transversality conditions $\lambda_{jT}^{h}=V_{j}^{n}$ for *h* ∈ {*s*, *i*, *q*, *r*}.

Conditions (10a) and (10b) for the social optimum are formally equivalent to conditions (7) and (8) for the private optimum, except that the individual value of being in state *s* (or *i*) at time *t* + 1, $V_{j,t+1}^{s}$ (or $V_{j,t+1}^{q}$), is replaced by the social value of an extra individual in state *s* (or *i*) at time *t*, $\lambda_{jt}^{s}$ (or $\lambda_{jt}^{i}$). The calculus for determining the optimal number of physical social contacts is the same for the utilitarian planner as for an individual. The marginal utility of an extra contact is set equal to the marginal cost in terms of increased number of individuals becoming infected. The difference, however, is that the planner considers the social cost of one extra individual becoming infected, which is $\lambda_{jt}^{s}-\lambda_{jt}^{i}$, and different from the individual cost of becoming infected, $V_{jt}^{s}-V_{jt}^{i}$.

For dead or recovered individuals, there is no difference between social and individual values, as being dead or recovered does not effect the health of others. The social value of an extra dead is constant over time, $\forall t:\lambda_{jt}^{d}=\lambda_{j,t-1}^{d}=V_{j}^{d}$, and we thus obtain from (10e) that $\lambda_{jt}^{r}=V_{j,t+1}^{r}$ as well. Also for COVID-19 patients, there is no difference between social and individual values, as by assumption they are in strict quarantine and thus do not infect others, so that $\lambda_{jt}^{q}=V_{j,t+1}^{q}$.

The key difference between individual and social optimum is that (10c) differs from (4) in that the condition for the social optimum includes the effect of a change in the number of infected of type *j* on all susceptible individuals. If there are many susceptible individuals relative to infected individuals, this makes a substantial difference. Inserting $\lambda_{jt}^{q}=V_{j,t+1}^{q}$ in (10c), using the expression (6) for $V_{jt}^{i}$, and solving the recursive equation for $\lambda_{jt}^{i}-V_{j,t+1}^{i}$ establishes that the social cost of an infection in population group *j* at time *t* is given as the private cost of the infection minus the net present value of utility cost of reducing contacts from the individually optimal level *c*_*jt*_ to the socially optimal level $c_{jt}^{⋆}$ minus the net present value of the infection externality on all others:

$$\begin{array}{cc} \lambda_{jt}^{i} & =V_{j,t+1}^{i}-\sum_{\tau =t+1}^{T}{\left(\delta_{j}\;\left(1-\theta_{j}-\gamma_{j}^{i}-\alpha_{j}^{i}\right)\right)}^{\tau -(t+1)}\;\{(u_{j}^{s}(c_{j\tau })-u_{j}^{s}(c_{j\tau }^{⋆}))+\sum_{l}\;\delta_{l}\;\beta \;c_{l\tau }^{⋆}\;S_{l\tau }\;(\lambda_{l\tau }^{s}-\lambda_{l\tau }^{q})\}, \end{array}$$ (11)

where $V_{j,t+1}^{i}$ is the individual value of getting infected, where $\delta_{l}\;\beta \;c_{l\tau }^{⋆}\;S_{l\tau }\;(\lambda_{l\tau }^{s}-\lambda_{l\tau }^{i})$ is the current-value external effect of the infection on individuals of population group *l* at time *τ* and where $\delta_{j}\;\left(1-\theta_{j}-\gamma_{j}^{i}-\alpha_{j}^{i}\right)$ is the population group-specific discount factor. This discount factor depends on the subjective utility discount factor *δ*_*j*_ as well as the rates at which individuals get quarantined, recover, or die. Everything else equal, the external effect of an infection is smaller the more quickly it is detected and the individual is quarantined. In line with intuition, extensive testing to increase *θ*_*j*_ reduces the infection externality.

The last term on the right-hand side of (11) quantifies the well-known infection externality in a pandemic (also referred to as ‘health externality’). Society attaches a higher damage to an extra infection than the individual, as the last term on the right-hand-side of (11) is negative, since $\lambda_{lt}^{s}>\lambda_{lt}^{q}$ for all groups *l*.

### Individual behavior under imperfect altruism

We consider a large population, such that each individual’s contribution to welfare is negligibly small. Given that, we think of a perfectly altruistic individual as one who puts herself in the shoes of the social planner. The perfectly altruistic individual would thus choose her individual contacts such as to maximize the utilitarian welfare function that sums up the purely selfish part of utility of all individuals in society, and thus choose contacts according to (10a). In contrast, a purely selfish individual would choose contacts according to (8), as derived above.

An imperfectly altruistic individual is modeled as a hybrid between the two extremes. We model such behavior by the following equation stating that she would choose her physical social contacts ${\hat{c}}_{jt}$ according to

$$\begin{array}{c} {u_{j}^{s}}^{\prime }\left({\hat{c}}_{jt}\right)=\delta_{j}\;\beta \;I_{t}\;(\left(1-\varphi_{j}\right)\;(V_{j,t+1}^{s}-V_{j,t+1}^{i})+\varphi_{j}\;\frac{S_{jt}}{S_{jt}+I_{jt}}\;(\lambda_{jt}^{s}-\lambda_{jt}^{i})), \end{array}$$ (12)

where *φ*_*j*_ ∈ [0, 1] is the individual’s degree of altruism between zero, for the purely selfish individual, and one, for the perfectly altruistic individual. This captures the idea that a purely selfish individual considers only the individual expected cost of infection and thus behave as described by Eq (8). A perfectly altruistic individual, on the other hand, behaves like the social planner and thus behave as described by Eq (10a). An imperfectly altruistic individual takes into account both the individual and the social expected costs of an infection, such that the expected marginal costs of an infection considered by an imperfectly altruistic indivicual are a convex combination of the private and social costs. The parameter *φ*_*j*_ thereby can be considered a continuous measure of the decreee of altruism.

For a given degree of altruism, *φ*_*j*_, we can use (8) and (10a) to alternatively write

$$\begin{array}{c} {u_{j}^{s}}^{\prime }\left({\hat{c}}_{jt}\right)=\left(1-\varphi_{j}\right)\;{u_{j}^{s}}^{\prime }\left(c_{jt}\right)+\varphi_{j}\;{u_{j}^{s}}^{\prime }\left(c_{jt}^{⋆}\right), \end{array}$$ (13)

where *c*_*jt*_ are the purely selfish individual and $c_{jt}^{⋆}$ the utilitarian optimal contacts.

We further use *ψ*_*j*_ to denote the share of the marginal expected costs of social contacts that are due to the purely selfish motivation, that is, we write

$$\begin{array}{cc} {u_{j}^{s}}^{\prime }\left(c_{jt}\right) & =\psi_{j}\;{u_{j}^{s}}^{\prime }\left({\hat{c}}_{jt}\right). \end{array}$$ (14)

The remaining fraction 1 − *ψ*_*j*_ corresponds to the extra reduction effort the individual spends for others. In our calibration to Germany (see below), we use observations on ${\hat{c}}_{jt}$ and *ψ*_*j*_ in (14) to estimate the number of contacts a respondent would have chosen for purely selfish reasons.

### Bringing the model to data

To quantify and solve the model numerically for the utiliarian optimum and the Nash equilibrium with selfish or imperfectly altruistic individuals, empirical information is needed on the epidemiological parameters $\alpha_{j}^{i}$, $\alpha_{j}^{q}$ *β*, $\gamma_{j}^{i}$, and $\gamma_{j}^{q}$ for all groups *j*. One additionally needs not only the discount factors *δ*_*j*_, but in principle also the Bernoulli utility functions $u_{j}^{s}\left(c_{jt}\right)$, $u_{j}^{q}$, and $u_{j}^{r}$, and the present value an individual attaches to dying, $V_{j}^{d}$. Especially the information about these utility functions is difficult to obtain.

However, not all of these utility functions need to be specified for the purpose of this paper, due to the following result: To compute the Nash equilibrium and socially optimal distancing, all information required about utility in the health states of infected, quarantined, recovered, and dead is contained in the individual expected present value of becoming infected, $V_{jt}^{i}$. The reason is as follows. In the Nash equilibrium with private self-protection, the dynamics of physical social contacts is determined as the simultaneous solution, for all groups *j*, of the individual optimality conditions (8), the Bellman Eq (7) for $V_{jt}^{s}$, and the epidemiological dynamics (1). Once $V_{jt}^{i}$ is known for all *j*, these equations can be solved without separate information about $u_{j}^{q}$, $u_{j}^{r}$, or $V_{j}^{d}$.

Socially optimal contacts are determined by condition (10a), the time paths of the state and co-state variables in this equation, namely epidemiological dynamics (1), and equations (10b) and (11), along with initial and transversality conditions. The key issue is that (11) determines the social value of an infection as the sum of the individual expected present value of an infection $V_{jt}^{i}$—which includes the present values of subsequent health states—, the Nash equilibrium number of contacts *c*_*jt*_—which can be determined as described in the previous paragraph—and the value of the infection externality, which *does not* explicitly depend on the value of subsequent health states. Thus, the term $V_{jt}^{i}$ in (11) fully captures all information about $u_{j}^{q}$, $u_{j}^{r}$, and $V_{j}^{d}$ necessary to compute the social optimum.

Before explaining our method to calibrate $V_{jt}^{i}$ from cross-sectional survey data and rational expectations about the dynamics of the pandemic, we turn to the specification of momentary utility derived from physical social contacts, which we use in the empirical model. We assume that individuals have no systematic differences in their preferences over physical social contacts, and specify the utility function as

$$\begin{array}{c} u_{j}^{s}\left(c_{jt}\right)=\frac{1}{1-\varepsilon }\;(c_{jt}^{\varepsilon }-\varepsilon \;c_{jt}). \end{array}$$ (15)

With this specification, the ‘normal’ number of contacts, i.e. the utility-maximizing level of *c*_*jt*_ absent the pandemic, and the maximum of utility, are both normalized to one, $c_{jt}^{0}=\text{arg}\;\text{max}\;u_{j}^{s}\left(c_{jt}\right)=1$ and *u*_*j*_(1) = 1, independent of *ε*. Thus, all utility values, especially $V_{jt}^{q}$, are measured in units relative to the normal individual utility from contacts.

Our method to calibrate the individual value of an infection, $V_{jt}^{i}$, given a specification for *ε* and a calibration of discount factors *δ*_*j*_, is as follows. We use information about reported individual social distancing behavior, and postulate that individuals chose their physical social contacts according to the individual optimality condition (8). Due to forward-looking behavior, the expected marginal benefit of contact reductions depends on the individual’s expectation about the future dynamics of the epidemic. Our calibration approach accounts for this by assuming that individuals rationally expected the actual development. Between late March and summer 2020, the pandemic has been largely stabilized in Germany.

Our focus is this ‘first wave’ of the pandemic in Germany, in spring and summer 2020. The individual probability of getting infected remained constant for several weeks after March 2020 when we observed behavior, as the estimated number of infected remained largely constant at about 50 per 100,000 individuals [46]. Also, between April and August 2020, the basic reproduction number $R_{0}$ has always been around one, with an average of $R_{0}=0.98$, a standard deviation of 0.33, and without any discernible trend [46]. Until of August 2020, there have been about 220,000 cases of COVID-19 in Germany, 275 per 100,000 individuals. That is, by August 2020, still more than 99% of the population were susceptible to an infection with the coronavirus.

For our calibration we thus suppose that forward-looking individuals expected in March 2020 that *I*_*t*_ would remain at the prevailing level *I*_0_, and that the pandemic was in a quasi-steady state where $V_{jt}^{s}$ and $V_{jt}^{i}$ have been constant. Using this to solve (7) for a constant $V_{j0}^{s}$, and subtracting $V_{j0}^{i}$ on both sides of the equation, we get

$$\begin{array}{cc} V_{j0}^{s}-V_{j0}^{i} & =\frac{u_{j}^{s}\left(c_{j0}\right)-\left(1-\delta_{j}\right)\;V_{j0}^{i}}{1-\delta_{j}\;(1-\beta \;c_{j0}\;I_{0})}. \end{array}$$ (16)

From condition (8) that determines the individually optimal number of contacts—which we observe from survey data–and using the specification (15) of momentary utility from social contacts in the susceptible state, as well as (16), we obtain the individual present value of an infection:

$$\begin{array}{c} V_{j0}^{i}=\frac{1}{1-\delta_{j}}\;(c_{j0}^{\varepsilon }-\frac{\varepsilon }{1-\varepsilon }\;\frac{1-\delta_{j}}{\delta_{j}\;\beta \;I_{0}}\;c_{j0}^{\varepsilon -1}+\frac{\varepsilon }{1-\varepsilon }\;\frac{1-\delta_{j}}{\delta_{j}\;\beta \;I_{0}}). \end{array}$$ (17)

The right-hand-side of (17) is fully specified by data (*c*_*j*0_, *I*_0_) and calibrated parameter values (*δ*_*j*_, *ε*). Next we present the data and calibration before we turn to the results that we obtain by using these calibrated parameter values for the full dynamic solution and analysis of the empirical model.

## Calibration for Germany

### Epidemiological parameters

We distinguish four population groups based on age and gender. With regard to age, we differentiate between respondents younger than 60 years (young) and those with an age of at least 60 years (old) as this threshold is also commonly used to classify between epidemiological high- and low-risk groups. In total, we consider the four groups of young men, young women, old men, and old women.

We use the daily number of new infections and COVID-19 fatalities in Germany reported by the German government’s central scientific institution in the field of biomedicine [47]. We calibrate group-specific estimates for the COVID-19 mortality rate, $\alpha_{j}^{q}$. In the baseline calibration, we assume that the baseline infection rate, *β*, and the recovery rate, $\gamma_{j}^{q}$, are identical for all groups *j*. This means that differences in infection rates are captured exclusively by differences in social physical contacts between groups. We assume that no individual dies or recovers from the disease before it is detected, i.e. we set $\gamma_{j}^{i}=\alpha_{j}^{i}=0$. Appendix contains S1 Table in S1 File with the resulting parameter values, as well as details and discussion of the estimation procedure.

### Survey data and calibration of utility parameters

For the key utility parameter required for the calibration—the individual present value of an infection—we use survey data that we elicited from a representative sample of 3,501 Germans from March 20 to 27, 2020, and combine this with estimates on discount factors from the literature. The survey respondents are representative for the German population in terms of gender, age, education, and income. We excluded 112 respondents that answered the survey in less [more] than 3 [60] minutes due to concerns regarding fast-clicking or inattention as well as 3 respondents with a diverse gender as this population group would be too small for our analysis. We pre-registered the survey at the AEA RCT Registry (https://doi.org/10.1257/rct.5573-1.1) The survey has been approved by the ethics committee of the University of Hamburg. Further details on the study are provided in the appendix in S1 File.

In Table 1, we report the main variables of interest for the overall sample as well as for each population group individually (see also S2 Table in S1 File).

**Table 1:** Descriptive statistics of relevant survey responses.

	All	Population Group							
		Young men (*j* = 1)		Young women (*j* = 2)		Old men (*j* = 3)		Old women (*j* = 4)	
Change in contacts (15-point Likert scale)	4.81	5.31	***	4.35	***	5.16	**	4.51	*
	(3.48)	(3.48)		(3.41)		(3.35)		(3.61)	
*Reason for defense efforts (in %)*									
To protect me	51.96	49.89	***	50.76	*	56.31	***	55.00	***
	(21.75)	(22.26)		(20.74)		(21.83)		(22.14)	
To protect family & friends	30.03	29.94		31.14	**	28.87		28.58	*
	(15.90)	(16.33)		(15.36)		(16.53)		(15.37)	
To protect others	18.01	20.18	***	18.10		14.82	***	16.43	**
	(14.37)	(16.24)		(13.39)		(12.97)		(12.92)	
*Expectations*									
Expected income change (15-point Likert scale)	6.98	6.97		6.44	***	7.85	***	7.45	***
	(2.41)	(2.49)		(2.50)		(1.89)		(2.10)	
P(get infected) (in %)	38.10	41.29	***	40.16	***	32.33	***	31.78	***
	(22.40)	(23.33)		(23.15)		(19.27)		(18.74)	
P(get slightly ill) (in %)	50.65	54.00	***	53.42	***	44.76	***	42.34	***
	(21.71)	(21.75)		(21.57)		(20.10)		(20.26)	
P(get in acute danger) (in %)	34.65	31.74	***	30.48	***	42.52	***	43.27	***
	(20.88)	(19.62)		(18.92)		(21.58)		(22.73)	
*Contacts wrt. regulation (in %)*									
Less than required	0.07	0.10	***	0.06		0.07		0.03	***
	(0.26)	(0.29)		(0.25)		(0.25)		(0.17)	
According to regulations	0.30	0.34	***	0.32	*	0.21	***	0.24	**
	(0.46)	(0.47)		(0.47)		(0.41)		(0.43)	
More than required	0.63	0.57	***	0.62		0.72	***	0.73	***
	(0.48)	(0.50)		(0.49)		(0.45)		(0.44)	
*General preferences*									
Patience	8.11	8.12		8.23	**	8.06		7.81	***
	(2.12)	(2.08)		(2.10)		(2.15)		(2.22)	
Observations	3501	1137		1312		561		491	

*Notes*: The table shows mean values and standard deviations in parentheses. Change in contacts was elicited with a logarithmic Likert scale as described in the main text. Expected income changes from 2019 to 2020 were elicited using a 15-point Likert scale ranging from 1 (reduction to 10 percent) to 15 (tenfold increase) with a value of 8 representing unchanged income. Patience was elicited using the Likert scale question from [48]. Stars indicate the significance of the mean values for the respective group to the mean over all groups (t-tests).

* *p* < 0.1,

** *p* < 0.05,

*** *p* < 0.01

To elicit behavioral responses and to quantify reductions in physical social contacts (*c*_*jt*_), we asked respondents: *“Compared to the same week last year, by what percentage have you reduced or increased your physical, social contacts this week?”*. In the survey, we defined *“physical, social contacts”* as situations in which the respondent came closer than two metres to others. We collected responses on a 15-point log-scale ranging from *“reduction to zero”* to *“increasing by 10%”* which corresponds to a range of *c*_*jt*_, relative to normal, in the interval [0;1.1]. Converting the responses to actual values, the mean response corresponds to a frequency of physical social contacts of ${\hat{c}}_{jt}=0.25$ relative to normal. We observe some heterogeneity between population groups (see Table 1).

Our survey provides some evidence that respondents behave in an imperfectly altruistic manner. From another question in the survey, we know that defense measures can only in part be attributed by pure selfish behavior. Specifically, we asked: *“As far as you reduce physical, social contacts or take protective efforts such as intensive hand washing, in what proportions (in percentage points that sum up to 100%) do you do this in order to (i) Protect yourself and members of your household [x%]; (ii) Protect your family and close friends [y%]; Protect other people [100-x-y%]”*.

We observe that respondents, on average, attach a weight of only 52 percent to protect themselves when considering private defense measures. Thus, a considerable share of the reduction in contacts is not attributable to pure selfish behavior, but is due to impure altruistic motives, relating to the protection of family members and close friends (with a mean weight of 30 percent), as well as to others (18 percent). Although the motivation to contribute to the public good does not differ across gender, respondents older than 60 years attach a significantly higher ‘selfish’ weight on themselves when considering defense efforts. While young women (men) attach a weight of 49.2 (50.1) percent on impure altruistic motives, this altruistic weight is only 45.0 (43.7) percent for old women (men).

Besides the intrinsic motivation to engage in defense measures, external factors like governmental regulations could also affect private defense measures and potentially crowd out some of the intrinsic motivation (see, e.g., [49]).

We test for this by comparing differences in responses for those who participate in the survey before and after a contact ban for Germany has been announced on Sunday, March 22, 2020. While the announcement took place roughly in the middle of our data collection period, this leaves approximately half of the respondents unaffected by the contact ban, and at least some share of the week in question subject to regulation for the other half. We report the results of this analysis below, which indicates that the contact ban had no discernible effect on either defense measures or impure-altruistic motives.

As for the momentary utility derived from physical social contacts, we use the functional form (15). Whereas the utility-maximizing contacts and the corresponding utility level are independent of the specification of *ε*, the exact value of *ε* determines the marginal utility of contacts for *c*_*jt*_ < 1. The smaller *ε*, the higher marginal utility, i.e. the more strongly an individual wants to maintain at least some physical social contacts. In particular, for *ε* > 1, marginal utility is bounded for *c*_*jt*_ → 0, whereas it is infinite for *ε* ≤ 1 when *c*_*jt*_ → 0. We specify a value moderately below one, i.e. *ε* = 0.7, which implies that a social planner would never choose complete isolation *c*_*jt*_ = 0. The results are robust against alternative specifications of *ε*, except if *ε* > 1. For a specification *ε* > 1, marginal utility of contacts is bounded, and a complete isolation (or lockdown), *c*_*jt*_ = 0, becomes optimal.

To estimate *c*_*jt*_ for the period of the survey, we use reported changes in the number of physical social contacts in the past week (variable “Change in contacts”, see Table 1). We interpret these as the optimal number of social contacts, ${\hat{c}}_{jt}$, an imperfectly altruistic individual would choose. We map the original responses, recorded on a 15-point Likert scale ranging from “reduction to zero” to “increase by 10%” to numerical values, interpolating the non-specified values. We further use the reasons for defense efforts (variable “To protect me”, see Table 1) as an estimate for *ψ* defined in (14). From this we use the specification (15) in (14) to estimate the number of contacts a respondent would have chosen for purely selfish reasons as $c_{j0}={\left(1+\psi_{j}\;({\hat{c}}_{j0}^{\varepsilon }-1)\right)}^{1/\varepsilon }$. The observations for ${\hat{c}}_{j0}$ and the estimates for the choice of physical social contacts under purely selfish behavior *c*_*j*0_ are shown in the appendix (S1 Fig in S1 File). For ${\hat{c}}_{j0}$, i.e. the observed imperfect altruistic behavior, the mean is 0.25 and for *c*_*j*0_, i.e. the estimated purely selfish behaviour, the mean is 0.33. Mean reductions are thus to about a third of normal and reductions are more pronounced for altruistic behaviour.

Our calibration of the discount factors *δ*_*j*_ is based on evidence from the literature. As we did not find evidence for substantial differences in reported patience, we assume identical discount factors for all groups. For Germany, [50] estimate a median discount rate of 27.5 to 30 percent from an incentivized elicitation of time preferences. In following our revealed preference approach, we rely on these best available estimates of individual utility discount rates, but note that these are orders of magnitudes higher as compared to social utility discount rates as used by governments or recommended by economic experts [51]. For our main calibration we take a central estimate from the literature and use a 30 percent annual discount rate, corresponding to a discount factor of *δ* = 1.3^−1/52^ = 0.995 per week. We show below that our results are not sensitive to substantially different assumptions on time preference rates.

We use the data on individual distancing behavior, as well as the calibrated discount factors and epidemiological parameters, to estimate, by means of ordinary least squares, the individual present value of getting infected, $V_{jt}^{i}$, using (17), derived in section, for the four groups. Results are reported in S3 Table in S1 File. Generally we observe substantial heterogeneity of values within population groups, indicated by relatively large standard deviations. Moreover, distributions are skewed, as for most groups the (absolute value) median is much larger (smaller) than the mean. As theory predicts (see section), the individual damage of an infection should increase with the COVID-19 mortality risk. Consistent with the pattern of COVID-19 mortality rates, we find that the individual cost of being infected is larger for old than for young men and it is larger for old than for young women. The theory also predicts that the individual damage of an infection increases if $u_{j}^{i}$ is small, which is the case especially for risk averse individuals. We find that the individual dis-utility of an infection is larger for women than for men, which is consistent with the observation that women are less willing to take health risks than men.

## Results

We present the quantitative results for Germany in three steps. First, we focus on the utilitarian optimum. Here, we compute socially optimal epidemiological dynamics starting at the initial infection rates mid March 2020, i.e. at the time of our survey, and then vary initial infection rates to study how socially optimal frequency of physical social contacts depends on the number of infected. Second, we compare these results to equilibrium dynamics with private self-protection by purely selfish individuals. Third, we focus on the social distancing behavior of imperfectly altruistic individuals.

We set the time horizon to *T* = 72 weeks. In line with our focus on the first wave of the pandemic in Germany, we also focus our presentation of results on weeks 0 to 20, i.e. March to August 2020.

We implement our dynamic optimization model, and the solution of equilibrium dynamics, in the state-of-the art nonlinear programming solver Knitro (version 11.0) with AMPL [52, 53], commonly used in other fields of economics [54, 55]. In all numerical computations we found a unique Nash equilibrium. Details on the solution method and programming codes are provided in the Appendix in S1 File and downloadable in the online supporting information.

### Utilitarian optimum

**Fig 1:**
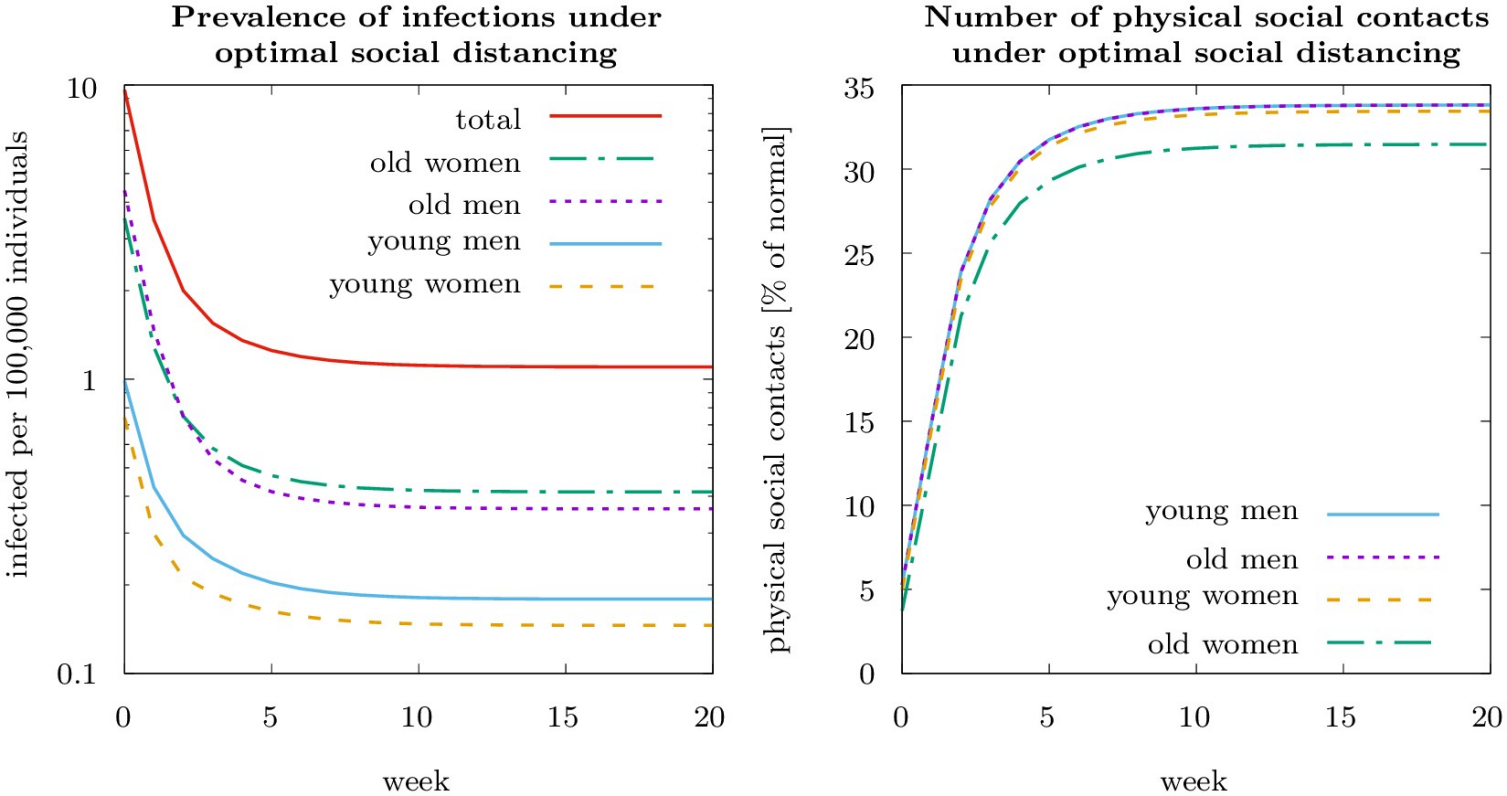
Dynamic optimization results for the first wave of COVID in Germany (starting March 2020). The left-hand panel shows the prevalence of infections for the four differnt groups and the total number of infections per 100,000 individuals. Parameter values as specified in the main text.

Fig 1 shows the socially optimal epidemiological dynamics, starting at the initial infection rates in Germany in mid March 2020, and the corresponding social distancing policy. Infection numbers follow a U-shaped pattern. It is optimal to drastically reduce infection numbers at the beginning, so that the disease is close to eradicated, with less than one infected per 100, 000 individuals (cf. Fig 1, left-hand panel. When considering the numbers of infected, it should be kept in mind that during the time when our data was collected, testing was still not quite as common as by the end of 2020.) Infection numbers are then optimally kept well below one infected per 100, 000 individuals. To attain this optimal trajectory, contacts are drastically reduced initially compared to pre-pandemic numbers, and during the quasi-steady state they are kept stable at about 33 percent of normal (see Fig 1, right-hand panel). These numbers of physical social contacts correspond to a basic reproduction rate of one, $R_{j0}=1$, i.e. one infected, on average, infects another individual. Differences in the number of contacts across groups are negligibly small.

**Fig 2:**
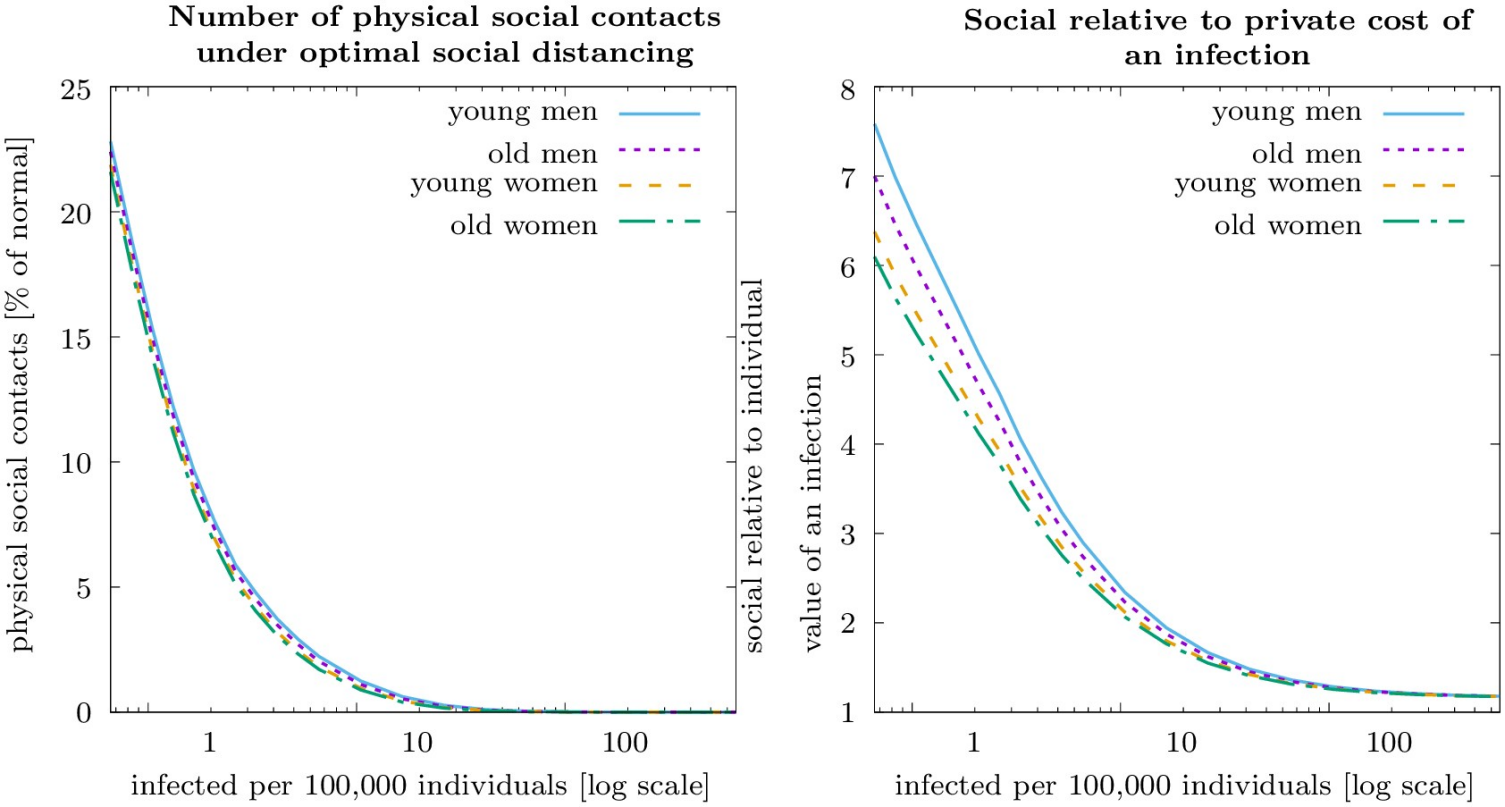
Optimal social distancing policy (left-hand panel) and social cost relative to private cost of infection, $\lambda_{jt}^{i}/V_{jt}^{i}$ (right-hand panel) as a function of current infected for the four different groups. Parameter values as specified in the main text.

A question of particular interest is, how the socially optimal distancing policy depends on the initial number of infected. The left-hand panel of Fig 2 shows that the optimal social distancing policy is a decreasing, convex function of current infection numbers. Already at one infected per 100, 000 individuals it is optimal to reduce physical social contacts to about 10 percent of the pre-pandemic level. At 10 infected per 100, 000 individuals contacts are reduced to about one percent and at around 100 infected per 100, 000 individuals a nearly complete lockdown is optimal. Differences in contact reduction between population groups are negligibly small relative to the contact reductions over the pre-pandemic level, with the first order effect being the response to infection numbers.

Social costs relative to private costs, $\lambda_{jt}^{i}/V_{jt}^{i}$, are particularly high at low infection numbers (cf. Fig 2, right-hand panel). At one infected per 100, 000 individuals the social costs is about five times higher than the private costs. The ratio of social relative to the private costs is decreasing with current infection numbers, reflecting that the individual risk of an infection increases relative to the external effect. This shows that the higher the private risk, the more would risk-averse, rational individuals contribute to the public good of preventing the epidemic from spreading. To study this in more detail, we next compare equilibrium dynamics with purely selfish individuals to the utilitarian optimum.

### Equilibrium dynamics with selfish individuals versus utilitarian optimum

**Fig 3:**
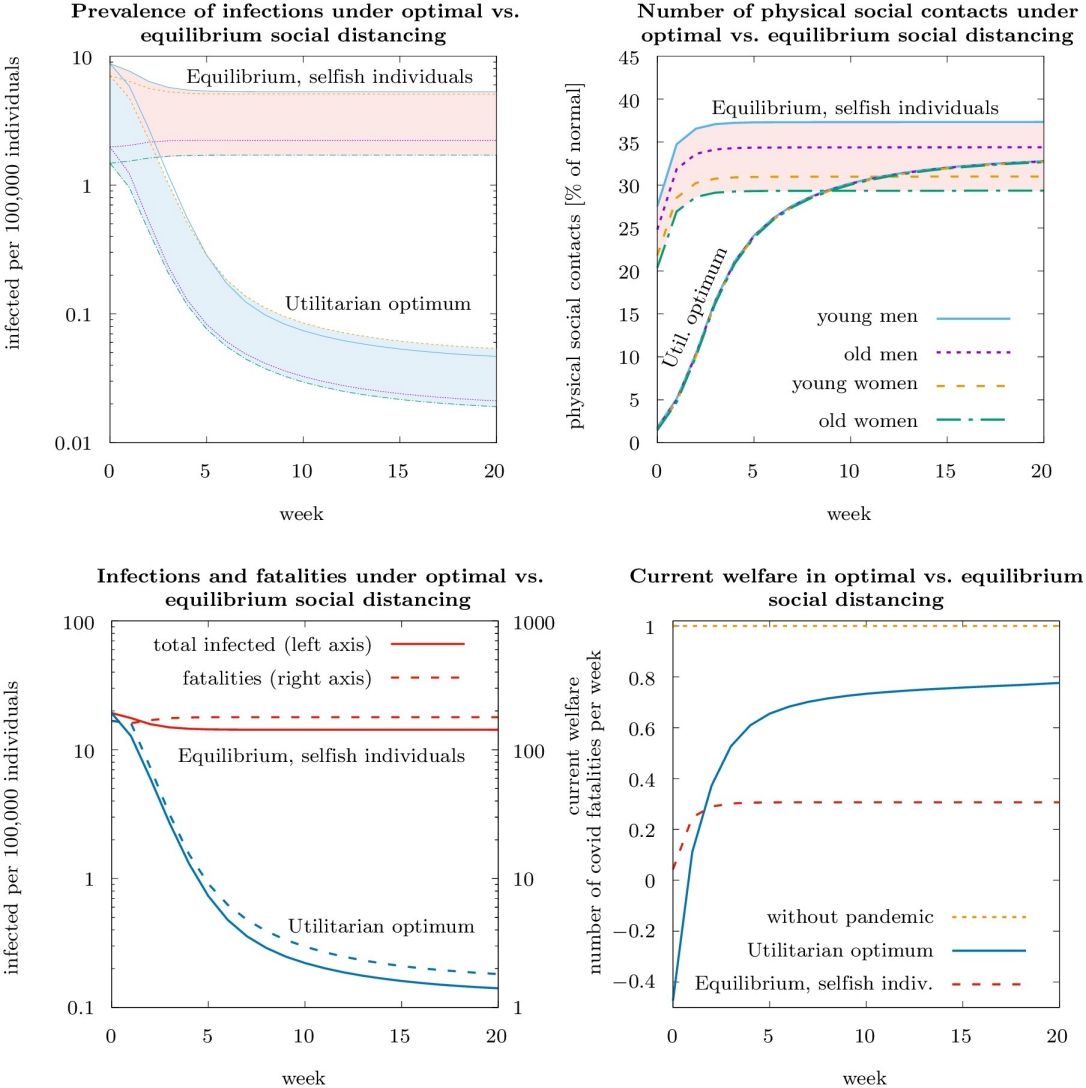
Epidemiological dynamics and individually optimal physical social contacts in Nash equilibrium of purely selfish individuals and under optimal social distancing policy (optimal dynamics as shown in Fig 1). The graphs on the top show the number of infections (top left-hand panel), the number of physical social contacts (top right-hand panel). The shaded areas display the respective spreads over the four groups of individuals. The graphs on the bottom show total prevalence of infections and the fatalities (bottom left-hand panel) and the current welfare (bottom right-hand panel). Parameter values as specified in the main text.

Fig 3 compares the epidemiological dynamics (infected per 100, 000 individuals, top left-hand panel) and contacts (as percent of normal) for (a) the open-loop Nash equilibrium of purely selfish individuals and (b) the utilitarian optimum, i.e. the same as shown in Fig 1. In Nash equilibrium, the reduction in contacts is initially much smaller than optimal. Also in Nash equilibrium a quasi-steady state is reached, and contacts are reduced to the level that keeps the basic reproduction rate of the epidemic at one, about a third of normal. This suggests that the selfish interest of rational, risk averse individuals to protect themselves from the disease may be sufficient to contain the virus. However, infection numbers that induce selfish individuals to self-protect to an extent that prevents the pandemic from spreading is about two orders of magnitude higher than in the social optimum. As a result, the number of people who die from COVID-19 in the Nash equilibrium is multiple times higher than in the optimum (Fig 3, bottom panel). Regarding differences across groups (Fig 3, top right-hand panel), in Nash equilibrium, young men have most contacts, followed by old men, following the individual valuation of an infection, as shown by our theoretical results. The effect that more individuals who are more severely affected by the disease impose less risks on others, which according to our theory plays the more important role in the social optimum, is irrelevant for equilibrium dynamics.

The bottom right-hand panel in Fig 3 shows the current welfare in Nash equilibrium with selfish individuals and the Utilitarian optimum. Welfare, normalized to one in the absence of a pandemic, is much smaller with the pandemic in place. This holds especially at the beginning of the pandemic when, in addition to the expected costs of an infection, the utility from physical social contacts is substantially reduced. As this effect is much more pronounced in the Utilitarian optimum, there is an initial phase where welfare is *smaller* in the Utilitarian optimum than in the Nash equilibrium with selfish individuals. This phase is an investment in future welfare gain through significantly reduced risk of an infection. In present value over the 20-weeks time horizon considered, welfare is significantly higher in the Utilitarian optimum (about 11.5) than in the Nash equilibrium (about 5.5), with a present value of 19.0 without the pandemic. The difference in the present values of welfare in the Utilitarian Optimum and in the Nash equilibrium—the latter being 52% of the welfare in the Utilitarian Optimum—can be viewed as a measure of the welfare loss from non-cooperative activities in the Nash equilibrium. It quantifies the *social efficiency deficit* for the first wave of the pandemic in Germany [56–58].

### Social distancing behavior of imperfectly altruistic individuals versus selfish individuals versus utilitarian optimum

Table 2 compares the contact reductions at the beginning of the pandemic for three scenarios: The Nash equilibrium with purely selfish individuals, the Nash equilibrium with imperfectly altruistic individuals, and the utilitarian optimum. Selfish individuals would reduce their contacts already to between 29 percent (young women) and 40 percent (young men) of pre-pandemic levels. Altruistic behaviour, as observed in the survey, leads to even stronger contact reductions ranging from 21 percent (young women) to 29 percent (young men). This closes the gap between contact reductions in the social optimum and the purely selfish Nash equilibrium by around 30 percent.

**Table 2:** Number of physical social contacts (all in % of normal) in the different scenarios (for initial conditions as in Fig 1; mid March in Germany) and degree of altruism (in %).

		Young men (1)	Young women (2)	Old men (3)	Old women (4)
*Physical social contacts*					
utilitarian optimum	$c_{j0}^{⋆}$	2.59	2.22	5.22	3.51
selfish laissez-faire	*c*_*j*0_	40.00	29.27	36.82	30.70
altruistic (observed)	${\hat{c}}_{j0}$	29.17	20.73	27.30	22.77
altruistic contribution	$\frac{c_{j0}-{\hat{c}}_{j0}}{c_{j0}-c_{j0}^{⋆}}$	28.95	31.57	30.31	29.17
degree of altruism	*φ*_*j*_	7.80	9.34	11.78	10.23

**Fig 4:**
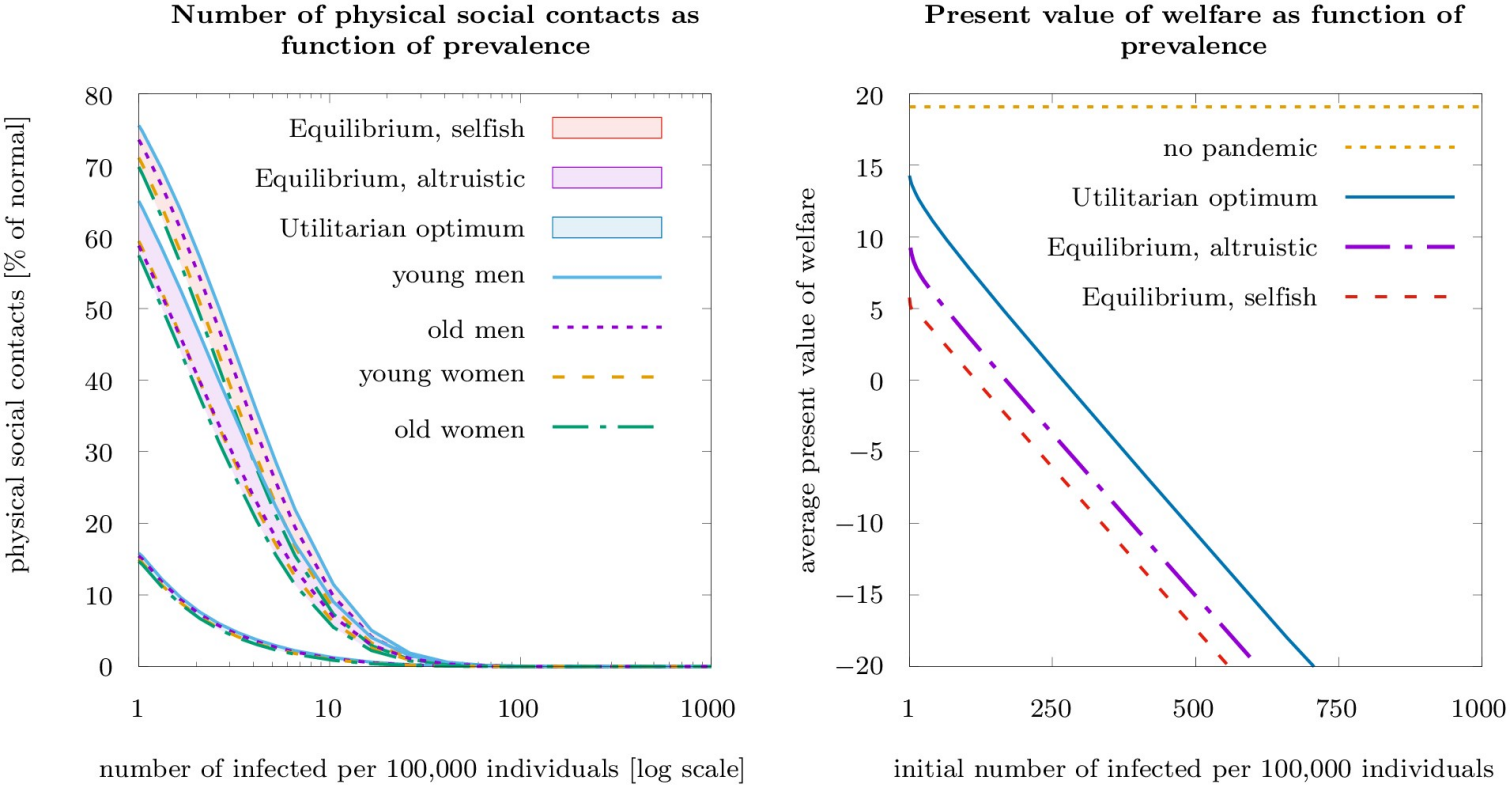
Physical social contacts (% of normal) depending on the number of infected in the Nash equilibria with purely selfish and imperfectly altruistic individuals, and in the utilitarian optimum (left-hand panel; logarithmic scale on the horizontal axis), and present values of welfare over 20 weeks in the three scenarios of utilitarian optimum and Nash equilibrium with imperfectly altruistic or selfish individuals (right-hand panel, linear scale on the horizontal axis).

Fig 4 (left-hand panel) compares the number of contacts for varying numbers of infected per 100, 000 individuals for the same three scenarios. The comparison shows that the difference between equilibrium and optimal distancing becomes small in absolute numbers if the number of infected gets large, as the substantial individual risk of infections is then sufficient to spur private contributions to the public good by risk-averse individuals. The difference between the frequency of contacts between the equilibrium, both with selfish and with imperfectly altruistic individuals, increases considerably as the number of infected decreases. In other words, policy intervention is particularly necessary when there are few infected individuals, whereas rational individuals will sufficiently self-protect and voluntarily contribute to the public good if the number of infected individuals is already large.

Fig 4 (right-hand panel) shows the loss in present value of welfare due to the pandemic as a function of the initial number of infected. The relationship is close to linear: An increase in the number of infected leads to a proportional extra welfare loss. The welfare difference between the Utilitarian optimum and the Nash equilibria with purely selfish or imperfectly altruistic individuals, respectively, (i.e., the social efficiency deficit in the terminology of [56–58]) is approximately constant, and insensitive to the initial number of infected. The increased reduction in the physical social contacts by altruistic compared to selfish individuals is reflected in a reduction of the social efficiency deficit: imperfect altruism closes about a third of the welfare gap between the Nash equilibrium with selfish individuals, on the one hand, and the Utilitarian optimum, on the other.

## Robustness checks and potential extensions

We have performed multiple further numerical studies to test the sensitivity of the results to key parameters, examine whether the contact ban has altered the voluntary reductions in contacts, and discuss how results might change with possible model extensions.

### Sensitivity analysis

The sensitivity analysis regarding epidemiological and preference parameters on the optimal dynamics in terms of the number of physical social contacts. Results are largely robust against all these alternative specifications. There is some sensitivity with respect to the parameters that determine the period of being infected without knowing, state *i*. If we assume that an infection is detected already after four days instead of five as in our baseline calibration, we get *θ* = 0.826. The result is that the quasi-steady state is reached more quickly and that slightly higher contact rates are admitted during the quasi-steady state. The reason is that at any point in time there are less infectious people in the population the more quickly infections are detected.

If we assume that infections are detected more quickly for old women and men than for young women and men (on average four days for old and six days for young individuals), which may be the case as old individuals more frequently develop symptoms, there is a moderate differentiation in contacts in the quasi steady state for the different groups. As our theory predicts, the old men and old women who are quarantined more quickly are allowed more social contacts than the young for whom it takes longer until they are quarantined. The difference is small compared to the overall reduction of contacts relative to normal, however.

As for the preference parameters, our baseline calibration assumes a weekly utility discount rate of 0.5 percent, corresponding to an annual discount rate of around 30 percent. The results are very robust against alternative specifications of the discount rate. Even for a very high discount rate of 5 percent per week, the general pattern of optimal dynamics remain similar, except that the quasi-steady state is approached more quickly. Also in the other extreme, if we set the discount rate to zero, results remain robust. We also compute optimal dynamics when individual expected present values of an infection would be at the median instead of mean values reported in S3 Table in S1 File, and if we re-calibrate these values assuming *ε* = 0.5 in the utility function (17), instead of *ϵ* = 0.7, as in the baseline calibration. The optimal policy is also robust against these alternative specifications of preference parameters.

Finally, we re-calibrate the model considering only a sub-sample of survey respondents. We turn to this in the next subsection.

### Effects of the contact ban and other distancing policies

**Fig 5:**
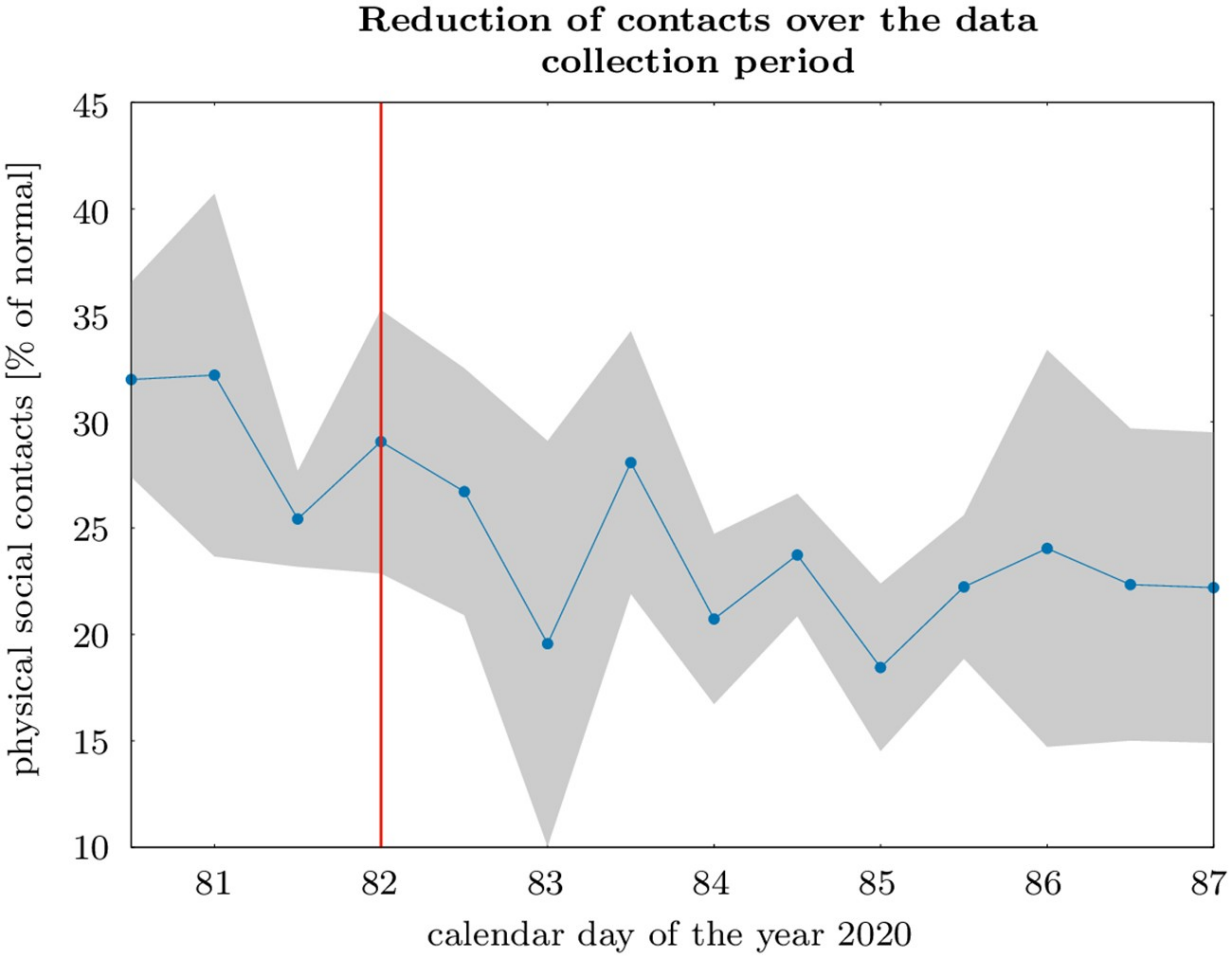
The graph shows the mean reduction in contacts, measured in percentage reduction from the same week of last year, grouped by day and daytime (before and after 12pm). The shaded area indicates deviations by two standard errors, and the red line the announcement of the contact ban in the evening of March 22, 2020.

During our data collection, the German government announced a nation-wide contact ban on March 22, 2020. This regulation did not allow meeting more than one other person at a time, except for members of the same household, but it did not constrain the total number of daily meetings. This regulation could have affected both the reduction in contacts and the motivation to engage in defense efforts. Table shows the result of the statistical test if there is a difference in the responses collected before and after the introduction of the contact ban. We do not find evidence that the contact ban affected the weights attached to the different reasons for individual protection efforts. Thus, we do not find any evidence for a crowding out of intrinsic motivation. With regard to the reported change of contacts during the past week, we observe a negative impact: after the contact ban, survey respondents tend to report stronger protection efforts, on average 0.442 points less on the 15-point Likert scale. However, we do not see a clear shift after the contact ban. To the contrary, we observe a continuous downward trend in contacts, as Fig 5 shows.

To study if our main results are affected by the different in social distancing behavior by early and late participants in the survey, we re-calibrate the model using data for the period March 20 to 22 only, ignoring all responses after the contact ban has been in force. This results in mean individual expected present values of an infection of $V_{1t}^{i}=-5,435$ for young men, $V_{2t}^{i}=-7,475$ for young women, $V_{2t}^{i}=-6,208$ for old men and $V_{4t}^{i}=-8,573$ for old women. The main effect is that the difference in mean values for men and women become more pronounced. Whereas the values for $V_{jt}^{i}$ for men in the early-respondent subsample are larger (smaller in absolute value) than for the whole sample, the values are smaller (larger in absolute values) for women. Overall the values are similar for both subsamples, however. Accordingly, the socially optimal frequency of physical social contacts is very similar for the re-calibrated model and for the baseline calibration using the full sample.

More generally, we consider the ‘selfish’ part of the individual reduction in the frequency of physical social distancing as the voluntary and unconstrained choice of the individual respondent, resulting in frequencies of contacts between 29.27 and 40.00 percent of normal (cf. Table 2). Our analysis has consistently shown that also in the Nash equilibrium with purely selfish individuals, eventually the epidemic will enter a quasi-steady state where individuals choose contacts ${\bar{c}}_{jt}$ such that their group-specific basic reproduction number would be equal to unity, $R_{j0}=\beta_{0}\;{\bar{c}}_{jt}/\left(\alpha_{j}^{i}+\gamma_{j}^{i}+\theta_{j}\right)=1$.

We now turn to the question how robust this result is and in particular to what extent it would be changed if the voluntary part of social distancing would be less (or more) than according to our estimates. A key parameter that we calibrate based on data on the observed physical social contacts (*c*_*j*0_) and prevalence of infections (*I*_0_) is the expected present value of an infection for an individual of type *i*, $V_{j0}^{i}$. We are interested in the question which other combinations of input data ${\bar{c}}_{j}$ and $\bar{I}$ would yield the same estimate for $V_{j0}^{i}$. We use equation (17) that determines the individual expected present value of an infection and compute the combination of ${\bar{c}}_{j}$ and $\bar{I}$ from the condition:

$$\begin{array}{c} \left(1-\delta_{j}\right)\;V_{j0}^{i}=c_{j0}^{\varepsilon }-\frac{\varepsilon }{1-\varepsilon }\;(c_{j0}^{\varepsilon -1}-1)\;\frac{1-\delta_{j}}{\delta_{j}\;\beta \;I_{0}}={\bar{c}}_{j}^{\varepsilon }-\frac{\varepsilon }{1-\varepsilon }\;({\bar{c}}_{j}^{\varepsilon -1}-1)\;\frac{1-\delta_{j}}{\delta_{j}\;\beta \;\bar{I}}. \end{array}$$ (18)

By rearranging the second and third part of (18), we obtain from this the share of infected individuals $\bar{I}$ as a function of ${\bar{c}}_{j}$ in quasi-steady state in the Nash equilibrium

$$\begin{array}{c} \bar{I}=\frac{\left(1-\delta_{j})\;\frac{\varepsilon }{1-\varepsilon }\;({\bar{c}}_{j}^{\varepsilon -1}-1\right)}{\delta_{j}\;\beta \;({\bar{c}}_{j}^{\varepsilon }-c_{j0}^{\varepsilon }+\frac{\varepsilon }{1-\varepsilon }\;(c_{j0}^{\varepsilon -1}-1)\;\frac{1-\delta_{j}}{\delta_{j}\;\beta \;I_{0}})}. \end{array}$$ (19)

**Fig 6:**
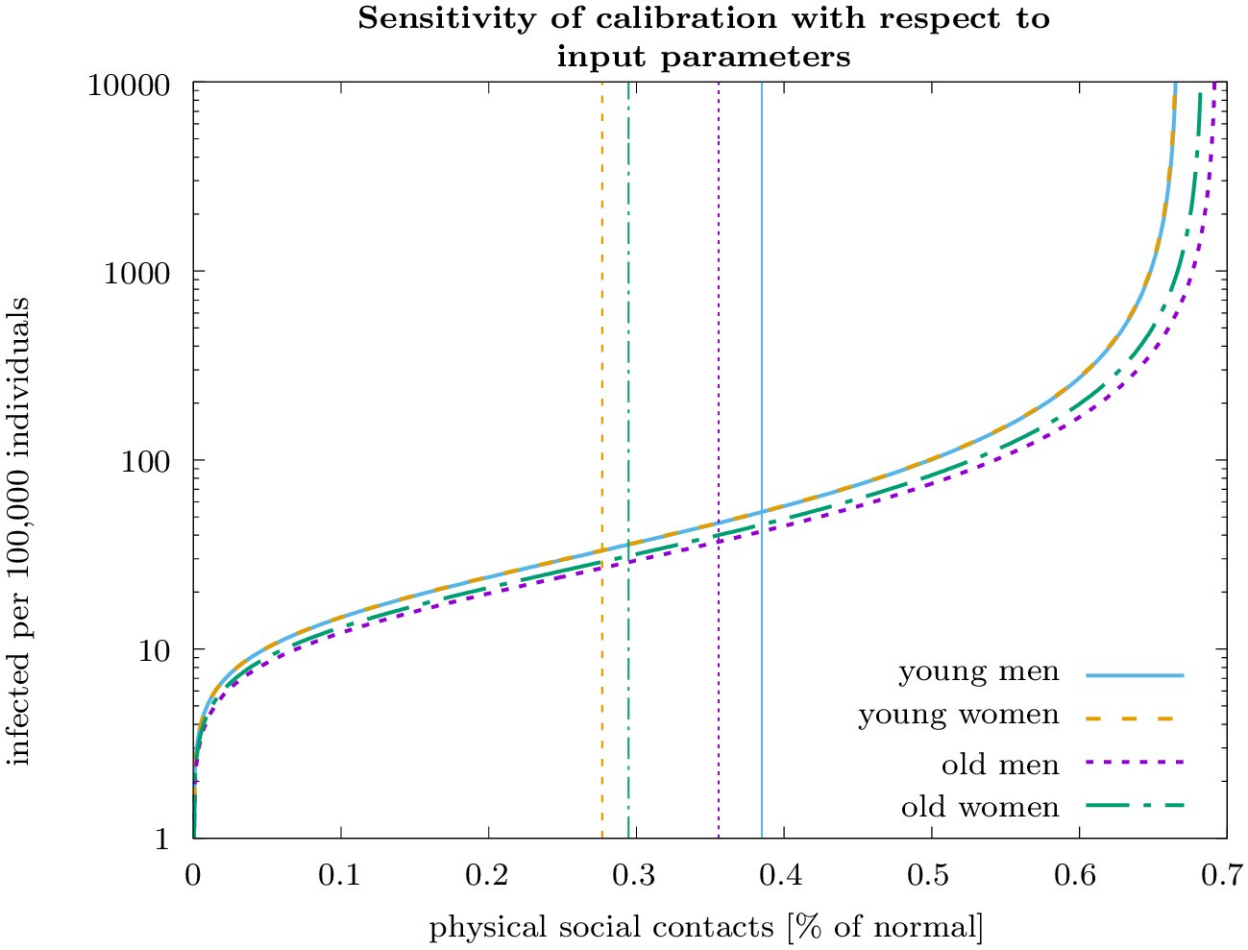
The figure shows which combinations of input data (physical social contacts and prevalence of infections, measured as infected per 100,000 individuals) give rise to the same calibration of the individual costs of an infection as the actual data we used (shown as vertical lines). We conclude that a broad range of plausible input data leads to a similar calibration.

Using the epidemiological data reported in S1 Table in S1 File, and the calibrated preference parameters *δ*_*j*_ and *ε*, we obtain the results shown in Fig 6.

This analysis shows that the calibration is robust over a wide range of alternative input data on the prevalence of infections and physical social contacts at the start of the pandemic. This is particularly true for prevalence rates between 10 and several hundred infected per 100,000 individuals, and for reductions in physical social contacts between 10% and 60%.

### Measuring physical distancing using cell-phone data

We finally compare the results of our calibrated model with movements from cell phone data. We use data from [59], which provides information on the number of cell phone movements at the county level in Germany (see [60] for an in-depth analysis). These movements capture switches in cell phone tower areas for users of the mobile phone providers Telekom and Telefónica, who account for a combined market share of around two-thirds [61]. In contrast to other studies that use mobility data from SafeGraph [12], Baidu [62], Apple [28], or Google, there are two major distinctions to highlight. First, the data we use are retrieved from mobile phone providers. Hence, they capture movements of cell phone users regardless of their installed apps, operating systems, or devices. Similarly, datasets on mobility patterns, as provided by Apple, Google, and Baidu, rely on the users of their navigation applications. Second, these cell phone movements reflect the number of trips instead of the number of devices at a specific location, like Point of Interest, or the time spend at home as provided by SafeGraph. While the latter is especially relevant for a US-style *shelter-in-place* policy, the German government introduced a contact ban but did not impose a nationwide curfew. Hence, the actual number of trips is the appropriate data to use for the case of Germany.

**Fig 7:**
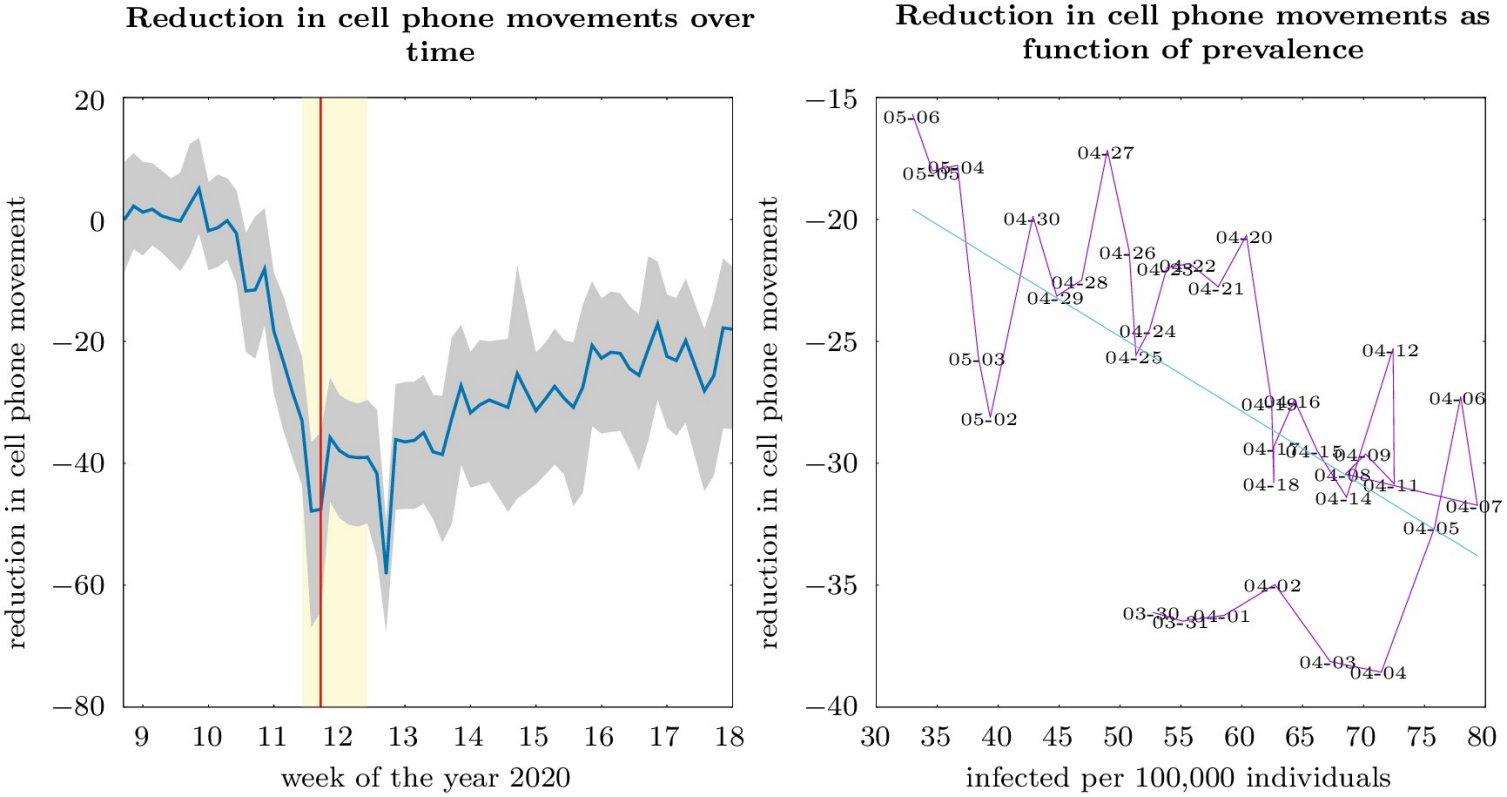
Reduction in cell phone movements in Germany during the COVID-19 spread the graphs show reduction in cell phone movements over a week(day) in Germany in 2020 relative to the average corresponding week(day) in 2019, excluding public holidays, aggregated to the county level (left-hand panel), and over the fraction of infected, starting on March 30, 2020 (week 13), where estimates of the number of recovered became available. In the left-hand panel, the blue line indicates the median county, the grey band the 10{90 percent interval, and the yellow band the time period of our survey. The red line indicates the contact ban announcement. Data is based on [59], using provider data from Telekom and Telefónica. A cell phone movement indicates a switch in cell phone tower areas after a person becomes stationary again.

Fig 7 (left-hand panel) shows cell phone movements in 2020 relative to the corresponding weekday in March 2019 over time. We observe are sharp reduction in cell phone movements of 40 to 50 percent starting from the beginning of March, but no clear reduction following the contact ban. During April, however, there is a steady convergence back to previous levels such that there are 20 percent fewer cell phone movements at the beginning of May. The right-hand panel in Fig 7 shows the same data, for the period March 30 to May 6, 2020, plotted over the estimated number of COVID-19 infections. Consistent with the model, cf. (8), there is a negative correlation between the reduction of cell phone movements, as a proxy for the reduction in the number of physical social contacts, and the number of infected individual.

### Discussion of potential extensions

As any model analysis, ours abstracts from a number of potentially interesting issues. In the following, we discuss potential extensions and the likely effects on results, based on the literature.

We do not consider limits to the health care system and thus a ‘health care externality’ in addition to the infection externality (see, e.g., [12, 15]), as it does not seem to be of practical relevance for our German case study. Yet, in principle, the model could be readily extended along these lines by making transition rates $\gamma_{j}^{q}$, and $\alpha_{j}^{q}$ dependent on *I*_*t*_. This would likely have the effect that the social cost of an elderly infection will rise, due to longer stay in hospitals. For instance, Farboodi et al. [12] find that considering in addition to the infection externality also a health care externality, i.e. that the quality of health care decreases as more individuals get infected due to capacity constraints and congestion of the health care system, leads to an even stronger ‘flattening of the curve’ in the social optimum.

Second, we did not consider issues of detecting infectious individuals and optimal testing. In the framework of our model this would mean to endogenize the rates *θ*_*j*_ at which infections are detected. Such a model could be used to study optimal population group-specific testing in a situation where testing is costly with increasing marginal costs. For instance, Brotherhood et al. [21] augment the SIR model to include an additional health state at which individuals show symptoms, but uncertainty about whether they have COVID-19 or a common flu only resolves after some time. Acemoglu et al. [63] model a situation where testing allows for a faster isolation of infectious individuals, but also increases contacts and thus can result in more infected.

Third, the model used here can facilitate any number of population groups and our empirical analysis can be extended accordingly. For instance one could consider age classes of 10-years, distinguish by income or pre-existing illnesses. We have limited our analysis to four population groups (distinguished by age and gender) that show significant differences in their contact reductions and in other key characteristics in our German data for expositional purposes. As the virological literature on more fine-grained differences across population groups is in flux, such extensions would be worthwhile retrospectively when sufficient clarity has been achieved.

Fourth, our models leaves aside a number of uncertainties about the evolution and the effect of COVID-19 that should be considered in follow-up work, such as when an effective vaccine becomes available, how long immunization holds for previously infected individuals, or virus mutations that change epidemiological parameters such as infectiousness and mortality.

Finally, we have taken a Utilitarian welfare function as the social objective, thereby following an approach common in both economics and moral philosophy. However, it would be interesting to compare this to alternative objectives, such as Prioritarianism [64], or approaches that specifically value individual freedom of movement or choice of contacts, for instance.

## Conclusion

Extending the epidemiological SIR model we have developed an economic-epidemiological model with forward-looking heterogeneous individuals susceptible to virus infection. Imperfectly altruistic subjects choose their number of contacts balancing current utility from physical social contacts with the expected present value of the infection risk. We have characterized private behavior of individuals susceptible to a virus infection, and socially optimal distancing. We have quantified the model with unique data on social distancing behavior and impure altruistic motivations from a large, representative survey among around 3,500 Germans conducted at the beginning of the COVID-19 pandemic, and we have calibrated our model to official epidemiological data for the first wave of the pandemic in spring and summer 2020 in Germany.

We find that the optimal policy would have reduced contacts drastically at the beginning of the pandemic to virtually eradicate the virus and to stabilize the spread at a quasi-steady state until a vaccine becomes tangible. Moreover, we find a substantial gap between private and social costs of contacts. The social costs of an infection are around twice as high as the private costs at the selfish Nash equilibrium, and more than five times higher at the socially-optimal level of infected. Pure selfish behavior does not lead to such a drastic initial reduction in contacts, but also reaches a quasi-steady state at infection levels of around 10 infected per 100,000 individuals. This is very moderate compared to a potential peak without behavioral adjustments, but far higher than in the social optimum. Moreover, the impure altruistic behavior of our respondents closes around one third of the gap between the selfish ‘laissez-faire’ and socially optimal contact reductions. Altruism also increases welfare compared to the selfish ‘laissez-faire’, but a gap still remains as selfish motives still play a more important role than altruism.

This adds new evidence to a long-standing literature by pointing towards an important role of impure altruism for the private provision of a public good—in an environment where both the externality and the number of people benefiting from a reduction in externality are very large [38–41, 65]. Overall, we find that although there is a considerable gap between the private and social costs of contacts, private measures for self-protection and the protection of others can contribute significantly to mitigating the problem of social costs. Our study thus also contributes with a high-stakes case study to the literature on the private provision of a public good under uncertainty [2, 34–37].

Of course, our study is subject to a number of limitations. First, our contact reduction survey responses are based on reported rather than observed behavior. However, the comparison with contacts based on mobile phone data showed that both approaches to observing social distancing behavior were broadly consistent. In addition, reported contact reductions may mitigate the effects of milder regulations prior to the contact ban through reduced contact opportunities, such as the cancellation of major events. Our survey data show that the majority of respondents in all groups reduce contacts more than necessary. So while it seems difficult to disentangle voluntary action from regulatory responses, our interpretation of the selfish Nash equilibrium may be too optimistic about what private action can do to help contain the pandemic. However, our analysis shows that our results are qualitatively robust to significant misinterpretations of this kind.

Secondly, we have not explicitly studied income losses due to social distancing during the pandemic. Rather, we assumed that individuals would include their contact-related income losses in their internal decisions for contact reduction, since our survey question did not distinguish between work-related or leisure-related contacts. Future work should include the utility depending on health status, (social) contacts and income from work contacts and examine to what extent the production depends on contacts and to what extent work can be carried out remotely [66, 67].

Finally, we have assumed that the marginal utility of physical social contacts is decreasing, implying that some types of contacts are more important than others. However, while individuals have a priority order of contacts and would choose their contracts differently from those prescribed by governments, contact bans have an additional loss of value compared to voluntary choice. Because our data does not unravel the different types of contacts, we cannot recommend what types of contacts should be prohibited or allowed, nor can we discuss possible “social contact budget” mechanisms, such as individually transferable quotas for contacts or liability rules.

One aim of this paper was to study what would have been the dynamic of the pandemic during the first wave in Germany under the assumption of purely selfish or imperfectly altruistic behavior. In the context of the COVID-19 pandemic, our results imply that the “flattening of the curve” observed in several Western societies, particularly in Germany, could be explained as a result of the Nash equilibrium outcome where imperfectly altruistic, risk-averse individuals choose distancing to protect themselves and others from an infection. While our data is for the German population, the model is generally applicable to all contexts where voluntary behavior during a pandemic plays a major role. This includes the United States where prevalence rates have been much higher than in Germany. Our model suggests that differences in individual risk preferences may (at least in part) explain differences in voluntary individual social distancing and thus differences in prevalence. Indeed, empirical evidence suggests that US Americans have a higher willingness to take risks than Germans [48], which provides an explanation why they also take higher individual health risks and thus contribute less to the protection of others as well.

While we can attribute most of the contact reductions observed in Germany to voluntary behaviour—in line with the evidence for COVID-19, for example by [49] and for A/H1N1 swine flu by [68]—the substantial gap between the prevalence of the disease in the Nash equilibrium and the social optimum clearly shows that government intervention remains necessary. In addition to strict contact regulations, public actors can play important roles informing about the risks, appealing to social norms [69], and making the infection risks salient.

## Supporting information

S1 Data (ZIP)

S1 File (PDF)
