# Supplementary material for: The social cost of contacts: Theory and evidence for the first wave of the COVID-19 pandemic in Germany
Source: PLoS One. 2021 Mar 19;16(3):e0248288. doi: 10.1371/journal.pone.0248288 (PMC7978372; doi:10.1371/journal.pone.0248288)
Supplement: S1 File — (PDF) [file pone.0248288.s002.pdf]

S1 File

S1 Appendix Derivations of theoretical results. The Lagrangian for the problem to optimize (9), subject to the epidemiological dynamics given by (1), reads

$$\mathbb{L} = \sum_j \sum_{t=0}^{T} \delta_j^t \Bigg\{ (S_{jt} + I_{jt})\, u_j^s(c_{jt}) + Q_{jt}\, u_j^q + R_{jt}\, u_j^r + D_{jt}\, u_j^d + \delta_j\, \lambda_{jt}^s \left(-S_{j,t+1} + S_{jt} - \beta\, c_{jt}\, S_{jt} \sum_l I_{lt} \right) + \delta_j\, \lambda_{jt}^i \left(-I_{j,t+1} + \beta\, c_{jt}\, S_{jt} \sum_l I_{lt} \right) + \delta_j\, \lambda_{jt}^q \left(-Q_{j,t+1} + (1 - \alpha_j^q - \gamma_j^q)\, Q_{jt} + I_{jt} \right) + \delta_j\, \lambda_{jt}^r \left(-R_{j,t+1} + R_{jt} + \gamma_j^q\, Q_{jt} \right) + \delta_j\, \lambda_{jt}^d \left(-D_{j,t+1} + \alpha_j^q\, Q_{jt} \right) \Bigg\}. \quad (20)$$

The first-order necessary conditions (10a) to (10f) for optimal social distancing are obtained by setting equal to zero the derivatives of the Lagrangian with respect to c_{jt} (equation 10a), S_{jt} (equation 10b), I_{jt} (equation 10c), Q_{jt} (equation 10d), R_{jt} (equation 10e), and D_{jt} (equation 10f), for all j and all t.

S2 Appendix Calibration of epidemiological parameters.

We estimate COVID-19 mortality rate α_j^q for each population group j as follows: We use the daily number of new known infections and new deaths in Germany reported by the Robert Koch Institute [1]. Here, we consider the time period from January 6 to April 26, 2020. and aggregate them to the weekly level. We use (1) and replace $\theta_j I_{jt}$ and $\alpha_j^q Q_{jt}$ with the time series of reported new known infections $\Delta Q_{j,t+1}$ and new deaths $\Delta D_{j,t+1}$ respectively. We suppose $Q_{j,1} = 0$, since the estimation relies on data $\Delta Q_{j,t}$, making $Q_{j,1}$ unimportant and because reported cases in January and early February were probably still affected by imported cases [2, 3]. We then estimate α_j^q by means of ordinary least squares

$$\min_{\{\alpha_j^q\}} \sum_{j=1}^{4} \sum_{t=1}^{15} \left(Q_{j,t}\, \alpha_j^q - \Delta D_{j,t+1} \right)^2, \tag{21}$$

subject to $Q_{j,t+1} = (1-\gamma)\, Q_{jt} + \Delta Q_{j,t+1} - \Delta D_{j,t+1}$. This gives α_j^q as shown in S1 Table.

Our estimate for θ_j is obtained from the time period until an infection is detected, on average, which we assume to be 5 days, which corresponds to an exponential detection rate of 1/5 days in a continuous time model. In our discrete time model the detection rate is $\theta_j = 1 - e^{-7/5} = 0.75$ per week. The initial basic reproduction number $\mathcal{R}_0$ was estimated to be between 2 and 3 [4–7], although higher values with a range of 3 to 12 [8] or up to 14.8 [9] have been suggested. We calibrate our model to the more conservative and widely accepted value of $\mathcal{R}_0 = 3$ for the German population during the initial phase of the pandemic. From this we obtain $\beta = 2.25$.

We calibrate γ_j^q such that 90% of COVID-19 patients recover within two weeks after the infection is detected. In our discrete time model the recovery rate is $\gamma_j^q = 1 - e^{-7/6}$ per week. The assumption of an exponential distribution captures the fact that some individuals need much longer to recover.

This implies the infection fatality ratios $\text{IFR}_j = \frac{\alpha_j^q}{\alpha_j^q + \gamma_j^q}$ as reported in S1 Table. The estimated mean IFR of 3.86 percent for the German population is close to the fatality rates reported by the WHO [10].

S1 Table Calibrated epidemiological parameters.

	All	Population group			
		Young men (j=1)	Young women (j=2)	Old men (j=3)	Old women (j=4)
α_j^q	0.0306	0.00285	0.000863	0.125	0.0849
β/N_0	2.25	2.25	2.25	2.25	2.25
γ_j^q	0.689	0.689	0.689	0.689	0.689
θ_j	0.75	0.75	0.75	0.75	0.75
IFR_j (%)	3.86	0.41	0.13	15.36	10.95

Parameters of the model for individuals in the respective population groups. All rates are in units of 1/week.

S3 Appendix Descriptive statistics of survey data.

Here we present some extra information about the data we used in our empirical model. S2 Table presents the summary statistics for the variables of interest. S1 Fig shows histograms of surveyed impure altruistic physical social contacts (left) and estimated contacts under purely selfish behavior (right) compared to normal. Overall the purely selfish contacts are higher, although still a substantial fraction reports a very low number of physical social contacts. S3 Table contains the full information about the individual costs of an infection for the different population groups, estimated by means of ordinary least squares. Table shows the result of the statistical test if there is a difference in the responses collected before and after the introduction of the contact ban. We do not find evidence that the contact ban affected the weights attached to the different reasons for individual protection efforts. Finally, S2 Fig shows a significant positive correlation between the reported physical social contacts from the survey and the mobility information derived from cell-phone data.

S2 Table Summary statistics of the survey data.

	N	Mean	Median	Std. Dev.	Min.	Max.
Change in contacts	3495	4.81	4	3.48	1	15
Age	3501	50.05	51	15.40	18	97
Female	3501	0.51	1	0.50	0	1
Family members and friends	3495	14.10	10	13.02	0	100
Reason for defense efforts (in %)						
To protect me	3499	51.96	50	21.75	0	100
To protect family & friends	3499	30.03	30	15.90	0	100
To protect others	3499	18.01	20	14.37	0	100
Expectations						
Expected income change	3494	6.98	8	2.41	1	15
P(get infected) (in %)	3486	38.10	40	22.40	0	100
P(get slightly ill) (in %)	3404	50.65	50	21.71	0	100
P(get in acute danger) (in %)	3404	34.65	30	20.88	0	100
Change wrt. regulation (in %)						
According to regulations	3501	0.30	0	0.46	0	1
Less than required	3501	0.07	0	0.26	0	1
More than required	3501	0.63	1	0.48	0	1
General preferences						
Patience	3499	8.11	9	2.12	1	11
Observations	3501					

S1 Fig. Histograms of surveyed impure altruistic physical social contacts (left), and estimated contacts under purely selfish behavior (right) compared to normal.

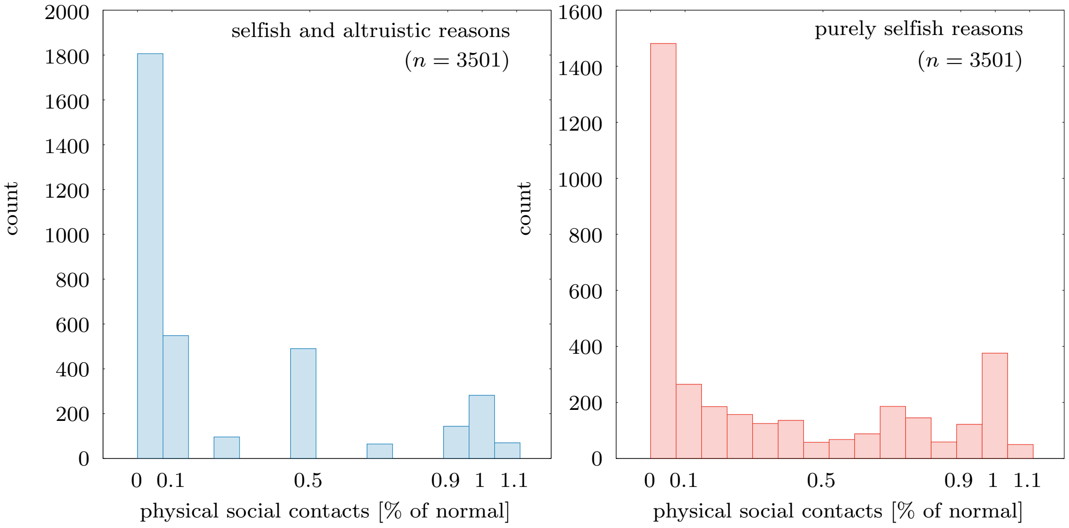

S3 Table Estimates for individual present values of getting infected for the four groups, V^i_{j0}.

	Young men (j=1)	Young women (j=2)	Old men (j=3)	Old women (j=4)
Mean	-5,780	-7,117	-6,360	-7,522
SD	5,201	5,480	5,840	6,113
Median	-3,620	-3,991	-3,631	-6,072

Momentary utility is measured relative to the maximum of utility derived from physical social contacts under normal conditions (absent pandemic), $\max_{c_{jt}} u^s(c_{jt}) = 1$. Present values are obtained applying the weekly discount factor for the baseline calibration, $\delta = 0.995$.

S4 Table. Effects of the contact ban announcement.

	Motivation			Contacts			
	Protect me (1)	Protect family & friends (2)	Protect others (3)	Past week (4)	Less than required (5)	As required (6)	More than required (7)
Post ban announcement	0.976	-0.875	-0.101	-0.442***	-0.016	0.086***	-0.070***
	(0.825)	(0.613)	(0.570)	(0.138)	(0.012)	(0.018)	(0.019)
Age	0.218***	-0.107***	-0.110***	-0.012**	-0.002***	-0.003***	0.005***
	(0.028)	(0.021)	(0.020)	(0.005)	(0.000)	(0.001)	(0.001)
Female	0.961	0.697	-1.658***	-0.966***	-0.054***	-0.027	0.081***
	(0.839)	(0.625)	(0.582)	(0.137)	(0.012)	(0.018)	(0.019)
Education							
University degree	-2.277	10.760*	-8.483	0.909	0.061	-0.018	-0.043
	(4.506)	(6.460)	(6.026)	(1.682)	(0.068)	(0.155)	(0.167)
A-levels/	-2.650	11.943*	-9.292	0.840	0.037	-0.051	0.015
vocational training	(4.478)	(6.449)	(6.007)	(1.682)	(0.068)	(0.155)	(0.167)
Secondary school	1.460	10.378	-11.838**	0.819	0.052	-0.054	0.002
	(4.442)	(6.435)	(5.987)	(1.679)	(0.068)	(0.154)	(0.167)
Secondary general school	0.473	10.688*	-11.161*	1.114	0.045	-0.032	-0.013
	(4.474)	(6.443)	(5.988)	(1.682)	(0.068)	(0.154)	(0.167)
Household income							
1,500 – 3,000	0.000	0.579	-0.579	0.013	-0.043**	-0.008	0.051*
	(1.203)	(0.879)	(0.771)	(0.201)	(0.019)	(0.027)	(0.028)
3,000 - 4,000	-0.940	1.044	-0.104	-0.277	-0.081***	-0.027	0.107***
	(1.286)	(0.949)	(0.877)	(0.221)	(0.019)	(0.030)	(0.031)
$\geq$ 4,000	0.350	0.201	-0.551	-0.624***	-0.057***	-0.070**	0.127***
	(1.433)	(1.040)	(0.949)	(0.233)	(0.021)	(0.031)	(0.033)
Observations	3481	3481	3481	3478	3483	3483	3483

Notes: OLS estimations for a weighted sample. Respondents before the ban are reweighted to match the mean values in age, gender, education, and income of those after the ban. Standard errors in parentheses. * $p < 0.1$, ** $p < 0.05$, *** $p < 0.01$

S2 Fig. Correlation between survey responses and cell phone movements.

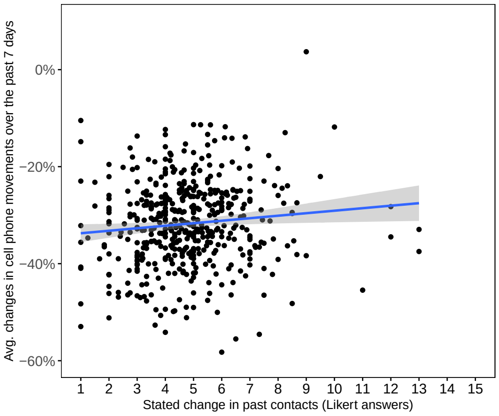

Plot shows the correlation between the average survey responses and reductions in cell phone movements per county.

S4 Appendix Numerical solution method and AMPL programming code

Numerically, the problem to solve the set of dynamic equations that describe epidemiological dynamics, and the optimality conditions, (1), (10a), (10b), and (11), along with initial conditions for the number of susceptibles, infected, and recovered from all groups of individuals, and transversality conditions, is usually a more difficult task than solving an optimization problem. In our numerical approach to compute the utilitarian optimum, we thus use the following lemma:

Lemma 1. *The social optimization problem* (9) *is equivalent to*

$$\hat{W} = \max_{\{c_{jt}\}} \sum_j \sum_{t=0}^{T} \delta_j^t \left((S_{jt} + I_{jt})\; u_j^s(c_{jt}) + I_{jt}\, \delta_j\, \theta_j\, V_{j,t+1}^q \right) \quad \textit{subject to } (1), \tag{22}$$

where $\bar{c}_{jt}$ is the individual choice of physical social contacts in Nash equilibrium and V_{jt}^q is the individual present value of becoming a COVID-19 patient, V_{jt}^q, as determined by (5).

Proof. The optimality conditions for (22) can be written as

$$u_j^{s\prime}(c_{jt}^\star) + \delta_j \frac{S_{jt}}{S_{jt} + I_{jt}}\, \beta\, I_t \left(\lambda_{jt}^i - \lambda_{jt}^s \right) = 0 \tag{23a}$$

$$u_j^s(c_{jt}^\star) - \lambda_{j,t-1}^s + \delta_j \left(1 - \beta\, c_{jt}^\star\, I_t \right) \lambda_{jt}^s + \delta_j\, \beta\, c_{jt}^\star\, I_t\, \lambda_{jt}^i = 0 \tag{23b}$$

$$u_j^s(c_{jt}^\star) + \delta_j\, \theta_j\, V_{j,t+1}^q - \lambda_{j,t-1}^i + \delta_j\, (1-\theta_j)\, \lambda_{jt}^i + \delta_j\, \theta_j\, \lambda_{jt}^q = \sum_l \delta_l\, \beta\, c_{lt}^\star\, S_{lt} \left(\lambda_{lt}^s - \lambda_{lt}^i \right) \tag{23c}$$

$$-\lambda_{j,t-1}^q + \delta_j\, (1 - \gamma_j^q - \alpha_j^q)\, \lambda_{jt}^q + \delta_j\, \gamma_j^q\, \lambda_{jt}^r + \delta_j\, \alpha_j^q\, \lambda_{jt}^d = 0 \tag{23d}$$

$$-\lambda_{j,t-1}^r + \delta_j\, \lambda_{jt}^r = 0 \tag{23e}$$

$$-\lambda_{j,t-1}^d + \delta_j\, \lambda_{jt}^d = 0, \tag{23f}$$

with transversality conditions $\lambda_{jT}^h = V_j^n$ for $h \in \{s, i\}$ and $\lambda_{jT}^h = 0$ for $h \in \{q, r, d\}$. The transversality conditions together with (23d), (23e) and (23f) imply $\lambda_{jt}^q = \lambda_{jt}^r = \lambda_{jt}^d = 0$ for all t in this auxiliary problem. Equations (23a) and (23b) are identical to (10a) and (10b). Using the above result $V_{j,t+1}^q = \lambda_{jt}^q$, equation (23c) is equivalent to (11). □

In the online Supporting Information we provide the AMPL code for computing the utilitarian optimal epidemiological dynamics, as shown in Figure 1.

To compute the Nash equilibrium with selfish individuals, we solve the system of equations (7), (8) and epidemiological dynamics (1) for $t = 0, \ldots, T$ with $T = 20$ weeks. This is done by means of a code similar to the one below, except that the objective function (9) is dropped (i.e. there is no opimization) and that equations (7) and (8) are imposed as conditions.

The number of contacts chosen by imperfectly altruistic individuals is computed from the optimal number of contacts and the number of contacts in the Nash equilibrium with selfish individuals, using these results in equation (13). Using the resulting number of contacts in (1), we simulate the resulting epidemiological dynamics and welfare in the Nash equilibrium with imperfectly altruistic individuals.

The codes used for computing the results for the other figures are all based on modifications of this code and are available upon request.

References

1. Robert Koch Institut. Fallzahlen in Deutschland; 2020. Available from: https://npgeo-corona-npgeo-de.hub.arcgis.com/datasets/dd4580c810204019a7b8eb3e0b329dd6_0.

2. Pinotti F, Di Domenico L, Ortega E, Mancastroppa M, Pullano G, Valdano E, et al. Lessons learnt from 288 COVID-19 international cases: importations over time, effect of interventions, underdetection of imported cases; 2020. Available from: https://www.medrxiv.org/content/10.1101/2020.02.24.20027326v1.full.pdf.

3. Rothe C, Schunk M, Sothmann P, Bretzel G, Froeschl G, Wallrauch C, et al. Transmission of 2019-nCoV infection from an asymptomatic contact in Germany. New England Journal of Medicine. 2020;382(10):970–971.

4. Boldog P, Tekeli T, Vizi Z, Dénes A, Bartha F, Röst G. Risk Assessment of Novel Coronavirus COVID-19 Outbreaks Outside China. Journal of Clinical Medicine. 2020;9(2):571.

5. Ferretti L, Wymant C, Kendall M, Zhao L, Nurtay A, Abeler-Dörner L, et al. Quantifying SARS-CoV-2 transmission suggests epidemic control with digital contact tracing. Science. 2020;368(6491).

6. an der Heiden M, Buchholz U. Modellierung von Beispielszenarien der SARS-CoV-2-Epidemie 2020 in Deutschland. Robert Koch Institut; 2020.

7. Read JM, Bridgen JR, Cummings DA, Ho A, Jewell CP. Novel coronavirus 2019-nCoV: early estimation of epidemiological parameters and epidemic predictions; 2020. Available from: https://www.medrxiv.org/content/10.1101/2020.01.23.20018549v2.

8. Maier BF, Brockmann D. Effective containment explains subexponential growth in recent confirmed COVID-19 cases in China. Science. 2020;368(6492):742–746.

9. Rocklöv J, Sjödin H, Wilder-Smith A. COVID-19 outbreak on the Diamond Princess cruise ship: estimating the epidemic potential and effectiveness of public health countermeasures. Journal of Travel Medicine,. 2020;forthcomming.

10. Baud D, Qi X, Nielsen-Saines K, Musso D, Pomar L, Favre G. Real estimates of mortality following COVID-19 infection. The Lancet infectious diseases. 2020;.
