# Supplementary material for: The social cost of contacts: Theory and evidence for the first wave of the COVID-19 pandemic in Germany
Source: PLoS One. 2021 Mar 19;16(3):e0248288. doi: 10.1371/journal.pone.0248288 (PMC7978372; doi:10.1371/journal.pone.0248288)
Supplement: Peer review history

# Peer review: Decision Letter 0

**DOI:** 10.1371/journal.pone.0248288.r001

**Authors:** Martial L Ndeffo Mbah

29 Jan 2021

PONE-D-20-38775

The Social Cost of Contacts:

Theory and Evidence for the first wave of the COVID-19 Pandemic in

Germany

PLOS ONE

Dear Dr. Quaas,

Thank you for submitting your manuscript to PLOS ONE. After careful consideration, we feel that it has merit but does not fully meet PLOS ONE’s publication criteria as it currently stands. Therefore, we invite you to submit a revised version of the manuscript that addresses the points raised during the review process.

Please submit your revised manuscript by Mar 14 2021 11:59PM. If you will need more time than this to complete your revisions, please reply to this message or contact the journal office at plosone@plos.org. When you're ready to submit your revision, log on to https://www.editorialmanager.com/pone/ and select the 'Submissions Needing Revision' folder to locate your manuscript file.

Please include the following items when submitting your revised manuscript:

- A rebuttal letter that responds to each point raised by the academic editor and reviewer(s). You should upload this letter as a separate file labeled 'Response to Reviewers'.
- A marked-up copy of your manuscript that highlights changes made to the original version. You should upload this as a separate file labeled 'Revised Manuscript with Track Changes'.
- An unmarked version of your revised paper without tracked changes. You should upload this as a separate file labeled 'Manuscript'.

If you would like to make changes to your financial disclosure, please include your updated statement in your cover letter. Guidelines for resubmitting your figure files are available below the reviewer comments at the end of this letter.

If applicable, we recommend that you deposit your laboratory protocols in protocols.io to enhance the reproducibility of your results. Protocols.io assigns your protocol its own identifier (DOI) so that it can be cited independently in the future. For instructions see: http://journals.plos.org/plosone/s/submission-guidelines#loc-laboratory-protocols

We look forward to receiving your revised manuscript.

Kind regards,

Martial L Ndeffo Mbah, Ph.D

Academic Editor

PLOS ONE

Additional Editor Comments:

There are several points that needs to clarify to improve transparency, readability, and enable an in depth assessment of the results. More details can be found within Reviewer 1 comments. Similar concerns are raised by Reviewer 3. Addressing these issue should greatly improve the quality of the manuscript and make it suitable for publication.

Journal Requirements:

When submitting your revision, we need you to address these additional requirements.

1. Please ensure that your manuscript meets PLOS ONE's style requirements, including those for file naming. The PLOS ONE style templates can be found at

https://journals.plos.org/plosone/s/file?id=wjVg/PLOSOne_formatting_sample_main_body.pdf and

https://journals.plos.org/plosone/s/file?id=ba62/PLOSOne_formatting_sample_title_authors_affiliations.pdf

2. Thank you for stating the following in the Acknowledgments Section of your manuscript:

"We acknowledge funding by the German Federal Ministry of Education and Research (BMBF) under

grant number 01LC1826E."

We note that you have provided funding information that is not currently declared in your Funding Statement. However, funding information should not appear in the Acknowledgments section or other areas of your manuscript. We will only publish funding information present in the Funding Statement section of the online submission form.

Please remove any funding-related text from the manuscript and let us know how you would like to update your Funding Statement. Currently, your Funding Statement reads as follows:

"The author(s) received no specific funding for this work."

Please include your amended statements within your cover letter; we will change the online submission form on your behalf.

3. Please ensure that you refer to Figures 2, 6, 9 in your text as, if accepted, production will need these references to link the reader to the figures.

[Note: HTML markup is below. Please do not edit.]

Reviewers' comments:

Reviewer's Responses to Questions

**Comments to the Author**

1. Is the manuscript technically sound, and do the data support the conclusions?

The manuscript must describe a technically sound piece of scientific research with data that supports the conclusions. Experiments must have been conducted rigorously, with appropriate controls, replication, and sample sizes. The conclusions must be drawn appropriately based on the data presented. 

Reviewer #1: Yes

Reviewer #2: Yes

Reviewer #3: Partly

**********

2. Has the statistical analysis been performed appropriately and rigorously? 

Reviewer #1: Yes

Reviewer #2: Yes

Reviewer #3: Yes

**********

3. Have the authors made all data underlying the findings in their manuscript fully available?

The PLOS Data policy requires authors to make all data underlying the findings described in their manuscript fully available without restriction, with rare exception (please refer to the Data Availability Statement in the manuscript PDF file). The data should be provided as part of the manuscript or its supporting information, or deposited to a public repository. For example, in addition to summary statistics, the data points behind means, medians and variance measures should be available. If there are restrictions on publicly sharing data—e.g. participant privacy or use of data from a third party—those must be specified.

Reviewer #1: Yes

Reviewer #2: Yes

Reviewer #3: Yes

**********

4. Is the manuscript presented in an intelligible fashion and written in standard English?

PLOS ONE does not copyedit accepted manuscripts, so the language in submitted articles must be clear, correct, and unambiguous. Any typographical or grammatical errors should be corrected at revision, so please note any specific errors here.

Reviewer #1: Yes

Reviewer #2: Yes

Reviewer #3: No

**********

5. Review Comments to the Author

Please use the space provided to explain your answers to the questions above. You may also include additional comments for the author, including concerns about dual publication, research ethics, or publication ethics. (Please upload your review as an attachment if it exceeds 20,000 characters)

Reviewer #1: This work concerns the epidemic SIR model, which poses social distancing for the COVID 19 pandemic in Germany. Authors try to find the gap between private and social cost of contact theoretically and verify it by using survey data. Although the work seems to contain some meaningful content from a theoretical and application point of view, I believe that several extra details should be introduced before considering the possibility of publishing this manuscript. Below I detail these concerns:

I think it will be significant if authors read and cite the newly established concept of the social efficiency deficit (SED). In the indicator SED, one can easily show the gap between social optimum and Nash equilibrium. They should read and introduce the idea of SED to show the gap (SED). It would be very interesting if the author can suggest how it will minimize this gap. Refer to the following kinds of literature to introduce the idea of dilemma strength,

Kabir, K. A., Tanimoto, J.; The role of advanced and late provisions in a co-evolutionary epidemic game model for assessing the social triple-dilemma aspect, JTB, 503, 110399 (2020).

Arefin, M.R., Kabir, K. M. A., Jusup, M., Ito, H., Tanimoto, J.; Social efficiency deficit deciphers social dilemmas, scientific reports, 10, 16092 (2020).

Kabir, KM. A., Tanimoto, J.; Modeling and analyzing the coexistence of dual dilemmas in the proactive vaccination game and retroactive treatment game in epidemic viral dynamics, Proc. R. Soc. A 475: 20190484 (2019).

The current form of all figures is unclear (axes and legends) and it is very difficult to understand the key point of your figures. I would like to ask you to redraw the figures with more details and give the details explanation in figure captions.

In the abstract, “Altruistic motives substantially increase individual protection efforts, but a substantial gap to the social optimum remains.”. This sentence seems to be very interesting and important to me. But I could not find a precise explanation for this, specifically in the result or conclusion section. Why this gap remains??

Reviewer #2: This model examines an SIR epidemic model of a population composed of multiple demographic groups, e.g. differentiation of age and gender, and dynamic social distancing strategies. The main goal of the analysis is to compare resulting societal welfare performance when distancing strategies are derived from a centralized social planner, versus decentralized selfish players. The model is quite complex with many parameters. The authors have gathered survey data from Germany in order to estimate and calibrate these parameters. The paper stipulates that selfish distancing behavior can stabilize the epidemic, but at a much higher infection cost than the optimal centralized policy. The presence of imperfectly altruistic individuals results in a balance between these two scenarios.

I find the paper is well written and precise. The derived conclusions are quite interesting. The mathematical model seems complex but is still numerically amenable with classic algorithms (dynamic programming). Hence, the model is quite valuable for offering predictions/insights for other scenarios that have not been as successful in containing COVID-19. For example in the U.S., where the rates are much higher than Germany, would the qualitative conclusions made in the paper still hold?

Regarding some technical aspects, it was not clear how the selfish and the semi-altruistic Nash equilibrium is computed. Also, it was not mentioned that there exists a unique equilibrium in both cases. If there are multiple Nash, then one would have to perform worst-case analysis, e.g. by calculating the price of anarchy.

It was also not clear how the the welfare function (eqn. 9) performs at the various Nash equilibria. The main figures 3 and 4 only show the effect on infection levels and contact levels. What would be interesting to see, which is not shown here, is the welfare as imperfect altruism ranges from completely selfish to completely altruistic. Is it obvious that this will be monotonically increasing? Surprising results can be found in other contexts, for example in congestion games:

Caragiannis, Ioannis, et al. "The impact of altruism on the efficiency of atomic congestion games." International Symposium on Trustworthy Global Computing. Springer, Berlin, Heidelberg, 2010.

Reviewer #3: Authors have computed utility functions of being socially distant considering individual vs. social cost using a discrete epidemiological model. They characterize the disparity in the Nash equilibrium and social optimum by quantifying it using Germany survey data on social distancing during COVID. The research is certainly valuable, important, timely and bears journal compatibility. However, I have preservation in presentation of the material in the draft. So, before it goes to publication house, I would like following things to be cleared. These are as follows pointwise:

1. The introduction is way more than expected by the reader. For example, authors have devoted approx. more than one and half page to portray how their work adds value compare to others. Reading these, it may loose some focus in the beginning of a draft. I would say shorten the introduction at least one page. Maybe some information to put in 'discussion' para.

2. The derivation of utility functions (2) - (6) should be explained in parts, better in appendix. Can authors use this reference to compare how they got or arrived those equations: Math Biosci. 2011 Apr; 230(2): 67–78. A general approach for population games with application to vaccination by Timothy Reluga and Alison P. Galvani. This is much more important to increase the audience of the paper.

3. Similarly, it is not clear how authors got the equations (10a-10f). Need more explanations and proper derivations.

4. How they got equation (12)?

5. Can author gives an justification why Bernoulli utility suits here in this epidemiological framework?

6. Parameter Estimation or calibration method is not clear from the text! Did they use some kind of MLE technique?

7. Neither clear how they arrived equation (18) and (19).

So, I mainly observe that paper lacks certain explanations, and derivations of mathematical equations. I encourage authors to revise the paper accordingly, and resubmit.

**********

6. PLOS authors have the option to publish the peer review history of their article (what does this mean?). If published, this will include your full peer review and any attached files.

If you choose “no”, your identity will remain anonymous but your review may still be made public.

**Do you want your identity to be public for this peer review?** For information about this choice, including consent withdrawal, please see our Privacy Policy.

Reviewer #1: No

Reviewer #2: No

Reviewer #3: No

[NOTE: If reviewer comments were submitted as an attachment file, they will be attached to this email and accessible via the submission site. Please log into your account, locate the manuscript record, and check for the action link "View Attachments". If this link does not appear, there are no attachment files.]

While revising your submission, please upload your figure files to the Preflight Analysis and Conversion Engine (PACE) digital diagnostic tool, https://pacev2.apexcovantage.com/. PACE helps ensure that figures meet PLOS requirements. To use PACE, you must first register as a user. Registration is free. Then, login and navigate to the UPLOAD tab, where you will find detailed instructions on how to use the tool. If you encounter any issues or have any questions when using PACE, please email PLOS at figures@plos.org. Please note that Supporting Information files do not need this step.

# Author comment: Author response to Decision Letter 0

**DOI:** 10.1371/journal.pone.0248288.r002

17 Feb 2021

We are grateful for the careful review and the invitation to revise and resubmit the manuscript. The guidance by the editor and the insightful review comments were very helpful in our major revision of the paper.

Our most important revisions have been the following:

1. We have included the concept of the social efficiency deficit, and added results on the welfare difference in the Utilitarian optimum and the Nash equilibrium with selfish and imperfectly altruistic individuals. 

2. We have added explanations and derivations of mathematical results. Among others, we now explain methods of computing the (unique) Nash equilibria and how we have calibrated the model.

3. We have updated the references, and added the extra references suggested by the reviewers. In the following we explain in detail how we have revised the manuscript in response to each of the reviewer comments. To this end, we reproduce the review reports and answer in blue italics. We have added numbering to the report by Reviewer 1 and Reviewer 2 to facilitate referencing.

Additional Editor Comments / Journal Requirements: "There are several points that needs to clarify to improve transparency, readability, and enable an in depth assessment of the results. More details can be found within Reviewer 1 comments. Similar concerns are raised by Reviewer 3. Addressing these issue should greatly improve the quality of the manuscript and make it suitable for publication."

*Response:* We thank the editor for the careful handling of the review process and the guidance for the revision. We have addressed all review comments, and feel that the manuscript greatly improved. We hope that you will find it suitable for publication now.

1. Please ensure that your manuscript meets PLOS ONE’s style requirements, including those for file naming. 

*Response:* We have prepared the revised manuscript accordingly.

2. Thank you for stating the following in the Acknowledgments Section of your manuscript: ...

*Response:* We have deleted the funding information in the Acknowledgments Section and included it in the submission system.

3. Please ensure that you refer to Figures 2, 6, 9 in your text as, if accepted, production will need these references to link the reader to the figures. 

*Response:* We have double checked the references to the figures.

Reviewer 1

Reviewer Comment: This work concerns the epidemic SIR model, which poses social distancing for the COVID 19 pandemic in Germany. Authors try to find the gap between private and social cost of contact theoretically and verify it by using survey data. Although the work seems to contain some meaningful

content from a theoretical and application point of view, I believe that several extra details should

be introduced before considering the possibility of publishing this manuscript. Below I detail these

concerns: 

*Response:* We thank the reviewer for the overall positive assessment of our contribution and for the constructive comments that helped us improving the manuscript.

Reviewer comment 1. I think it will be significant if authors read and cite the newly established concept of the social efficiency deficit (SED). In the indicator SED, one can easily show the gap between social optimum and Nash equilibrium. They should read and introduce the idea of SED to show the gap (SED). It would be very interesting if the author can suggest how it will minimize this gap. Refer to the following kinds of literature to introduce the idea of dilemma strength,

Kabir, K. A., Tanimoto, J.; The role of advanced and late provisions in a co-evolutionary epidemic game model for assessing the social triple-dilemma aspect, JTB, 503, 110399 (2020).

Arefin, M.R., Kabir, K. M. A., Jusup, M., Ito, H., Tanimoto, J.; Social efficiency deficit deciphers social dilemmas, scientific reports, 10, 16092 (2020).

Kabir, KM. A., Tanimoto, J.; Modeling and analyzing the coexistence of dual dilemmas in the proactive vaccination game and retroactive treatment game in epidemic viral dynamics, Proc. R. Soc. A 475: 20190484 (2019).

*Response:* We thank the reviewer for pointing us to this literature on the social efficiency deficit. We have included two new graphs showing the social efficiency deficit in Figures 3 and 4, and included a discussion and the suggested references in the presentation of these new results on pages 22 and 23.

Reviewer comment 2. The current form of all figures is unclear (axes and legends) and it is very difficult to

understand the key point of your figures. I would like to ask you to redraw the figures with more details and give the details explanation in figure captions. 

*Response:* We have augmented the legends and captions of the figures. Now it is much easier to understand the messages from the figures without having to go back to the main text.

Reviewer comment 3. In the abstract, “Altruistic motives substantially increase individual protection efforts, but a substantial gap to the social optimum remains.”. This sentence seems to be very interesting and important to me. But I could not find a precise explanation for this, specifically in the result or conclusion section. Why this gap remains??

*Response:* In the social optimum, in the action of each individual the effect on all others must be fully taken into account. Even for the altruistic individuals in our sample, the main motivation is egoistic, and the effect on others is only partly taken into account. This is why a gap remains. We have added a short explanation to the abstract and explain this in more detail now both in the results and conclusion section.

Reviewer 2

Reviewer comment: This model examines an SIR epidemic model of a population composed of multiple demographic groups, e.g. differentiation of age and gender, and dynamic social distancing strategies. The main goal of the analysis is to compare resulting societal welfare performance when distancing strategies

are derived from a centralized social planner, versus decentralized selfish players. The model is quite complex with many parameters. The authors have gathered survey data from Germany in order to estimate and calibrate these parameters. The paper stipulates that selfish distancing behavior can stabilize the epidemic, but at a much higher infection cost than the optimal centralized policy. The presence of imperfectly altruistic individuals results in a balance between these two scenarios.

*Response:* We thank the reviewer for the overall positive assessment of our contribution and for the constructive comments that helped us improving the manuscript.

Reviewer comment 1. I find the paper is well written and precise. The derived conclusions are quite interesting. The mathematical model seems complex but is still numerically amenable with classic algorithms (dynamic programming). Hence, the model is quite valuable for offering predictions/insights for

other scenarios that have not been as successful in containing COVID-19. For example in the U.S.,

where the rates are much higher than Germany, would the qualitative conclusions made in the paper still hold? 

*Response:* While our data is for the German population, the model is generally applicable to all contexts where voluntary behavior during a pandemic plays a major role. This includes the US. Our model suggests that in particular differences in individual risk preferences may explain differences in individual social distancing behavior and thus differences in prevalence. Indeed, empirical evidence suggests that the willingness to take risks is higher for US Americans than for Germans (Falk et al. 2018, now cited in the paper), which provides an explanation why they also take higher individual health risks and thus contribute less to the protection of others as well. We have included this discussion in the final section of the paper.

Reviewer comment 2. Regarding some technical aspects, it was not clear how the selfish and the semi-altruistic Nash equilibrium is computed. Also, it was not mentioned that there exists a unique equilibrium in

both cases. If there are multiple Nash, then one would have to perform worst-case analysis, e.g. by calculating the price of anarchy. 

*Response:* We have added the explanation how we computed the selfish and the semi-altruistic Nash equilibria in Appendix D. In all our numerical computations the Nash equilibrium was unique. We state this now explicitly at the beginning of the results section. 

Reviewer comment 3. It was also not clear how the the welfare function (eqn. 9) performs at the various Nash equilibria. The main figures 3 and 4 only show the effect on infection levels and contact levels. 

What would be interesting to see, which is not shown here, is the welfare as imperfect altruism ranges from completely selfish to completely altruistic. Is it obvious that this will be monotonically increasing? Surprising results can be found in other contexts, for example in congestion games: Caragiannis, Ioannis, et al. “The impact of altruism on the efficiency of atomic congestion games.” International Symposium on Trustworthy Global Computing. Springer, Berlin, Heidelberg, 2010. 

*Response:* We see the point that surprising results may happen in such contexts, and briefly discuss this in footnote 17. To explore if this happens in our analysis, we present new results on the present value of welfare in the different settings in the new graph in Figure 4. These results show that no such unexpected results occur in our model.

Reviewer 3

Reviewer comment: Authors have computed utility functions of being socially distant considering individual vs. social cost using a discrete epidemiological model. They characterize the disparity in the Nash equilibrium and social optimum by quantifying it using Germany survey data on social distancing during

COVID. The research is certainly valuable, important, timely and bears journal compatibility. However, I have preservation in presentation of the material in the draft. So, before it goes to publication house, I would like following things to be cleared. These are as follows pointwise: 

*Response:* We thank the reviewer for the overall positive assessment of our contribution and for the constructive comments that helped us improving the manuscript.

Reviewer comment 1. The introduction is way more than expected by the reader. For example, authors have devoted approx. more than one and half page to portray how their work adds value compare to

others. Reading these, it may loose some focus in the beginning of a draft. I would say shorten the introduction at least one page. Maybe some information to put in ‘discussion’ para. 

*Response:* We have now largely moved the discussion how our contribution relates to the previous literature to a new section. Thus we could shorten the introduction substantially, and readers not interested in the related literature can simply skip that new section.

Reviewer comment 2. The derivation of utility functions (2) – (6) should be explained in parts, better in appendix. Can authors use this reference to compare how they got or arrived those equations: Math Biosci. 32011 Apr; 230(2): 67–78. A general approach for population games with application to vaccination

by Timothy Reluga and Alison P. Galvani. This is much more important to increase the audience of the paper. 

*Response:* We have now included an explanation how our way of deriving utility functions – in a recursive fashion, using Bellman equations, relates to alternative approaches to formulate present value utilities such as the one used in Reluga and Galvani, now cited in the paper. See footnote 5 on page 7.

Reviewer comment 3. Similarly, it is not clear how authors got the equations (10a-10f). Need more explanations and proper derivations. 

*Response:* We have included an appendix where we present the explanations and proper derivations.

Reviewer comment 4. How they got equation (12)? 

*Response:* Equation (12) is a model assumption, capturing how we think about altruism. We explain this more carefully in the revised manuscript.

Reviewer comment 5. Can author gives an justification why Bernoulli utility suits here in this epidemiological framework? 

*Response:* The general theory of decision-making under uncertainty, which we apply here to the epidemiological framework, makes use of Bernoulli utility functions. We have included a corresponding explanation in footnote 6 on page 7.

Reviewer comment 6. Parameter Estimation or calibration method is not clear from the text! Did they use some kind of MLE technique? 

*Response:* We have used ordinary least squares. This is now mentioned explicitly in the main text (page 17) and in the appendix (page 33).

7. Neither clear how they arrived equation (18) and (19). 

*Response:* We see that the explanation around equations (18) and (19) was not quite clear. We have revised it thoroughly to make the point clearer.

Reviewer comment So, I mainly observe that paper lacks certain explanations, and derivations of mathematical equations. I encourage authors to revise the paper accordingly, and resubmit. 

*Response:* Many thanks again for the careful report and the overall positive assessment. We have added the required explanations and derivations and hope that you will find the revised paper suitable for publication.

# Peer review: Decision Letter 1

**DOI:** 10.1371/journal.pone.0248288.r003

**Authors:** Martial L Ndeffo Mbah

24 Feb 2021

The Social Cost of Contacts:

Theory and Evidence for the first wave of the COVID-19 Pandemic in

Germany

PONE-D-20-38775R1

Dear Dr. Quaas,

We’re pleased to inform you that your manuscript has been judged scientifically suitable for publication and will be formally accepted for publication once it meets all outstanding technical requirements.

Within one week, you’ll receive an e-mail detailing the required amendments. When these have been addressed, you’ll receive a formal acceptance letter and your manuscript will be scheduled for publication.

An invoice for payment will follow shortly after the formal acceptance. To ensure an efficient process, please log into Editorial Manager at http://www.editorialmanager.com/pone/, click the 'Update My Information' link at the top of the page, and double check that your user information is up-to-date. If you have any billing related questions, please contact our Author Billing department directly at authorbilling@plos.org.

If your institution or institutions have a press office, please notify them about your upcoming paper to help maximize its impact. If they’ll be preparing press materials, please inform our press team as soon as possible -- no later than 48 hours after receiving the formal acceptance. Your manuscript will remain under strict press embargo until 2 pm Eastern Time on the date of publication. For more information, please contact onepress@plos.org.

Kind regards,

Martial L Ndeffo Mbah, Ph.D

Academic Editor

PLOS ONE

Additional Editor Comments (optional):

Reviewers' comments:

Reviewer's Responses to Questions

**Comments to the Author**

1. If the authors have adequately addressed your comments raised in a previous round of review and you feel that this manuscript is now acceptable for publication, you may indicate that here to bypass the “Comments to the Author” section, enter your conflict of interest statement in the “Confidential to Editor” section, and submit your "Accept" recommendation.

Reviewer #1: (No Response)

Reviewer #3: All comments have been addressed

**********

2. Is the manuscript technically sound, and do the data support the conclusions?

The manuscript must describe a technically sound piece of scientific research with data that supports the conclusions. Experiments must have been conducted rigorously, with appropriate controls, replication, and sample sizes. The conclusions must be drawn appropriately based on the data presented. 

Reviewer #1: Yes

Reviewer #3: Yes

**********

3. Has the statistical analysis been performed appropriately and rigorously? 

Reviewer #1: Yes

Reviewer #3: Yes

**********

4. Have the authors made all data underlying the findings in their manuscript fully available?

The PLOS Data policy requires authors to make all data underlying the findings described in their manuscript fully available without restriction, with rare exception (please refer to the Data Availability Statement in the manuscript PDF file). The data should be provided as part of the manuscript or its supporting information, or deposited to a public repository. For example, in addition to summary statistics, the data points behind means, medians and variance measures should be available. If there are restrictions on publicly sharing data—e.g. participant privacy or use of data from a third party—those must be specified.

Reviewer #1: Yes

Reviewer #3: Yes

**********

5. Is the manuscript presented in an intelligible fashion and written in standard English?

PLOS ONE does not copyedit accepted manuscripts, so the language in submitted articles must be clear, correct, and unambiguous. Any typographical or grammatical errors should be corrected at revision, so please note any specific errors here.

Reviewer #1: Yes

Reviewer #3: Yes

**********

6. Review Comments to the Author

Please use the space provided to explain your answers to the questions above. You may also include additional comments for the author, including concerns about dual publication, research ethics, or publication ethics. (Please upload your review as an attachment if it exceeds 20,000 characters)

Reviewer #1: (No Response)

Reviewer #3: The paper looks good now with improved comments and additions. Authors have considered all comments and improved their paper. It is now acceptable for journal publication.

**********

7. PLOS authors have the option to publish the peer review history of their article (what does this mean?). If published, this will include your full peer review and any attached files.

If you choose “no”, your identity will remain anonymous but your review may still be made public.

**Do you want your identity to be public for this peer review?** For information about this choice, including consent withdrawal, please see our Privacy Policy.

Reviewer #1: No

Reviewer #3: **Yes: **SAMIT BHATTACHARYYA

# Editor report: Acceptance letter

**DOI:** 10.1371/journal.pone.0248288.r004

**Authors:** Martial L Ndeffo Mbah

9 Mar 2021

PONE-D-20-38775R1 

The Social Cost of Contacts: Theory and Evidence for the first wave of the COVID-19 Pandemic in Germany 

Dear Dr. Quaas:

I'm pleased to inform you that your manuscript has been deemed suitable for publication in PLOS ONE. Congratulations! Your manuscript is now with our production department. 

If your institution or institutions have a press office, please let them know about your upcoming paper now to help maximize its impact. If they'll be preparing press materials, please inform our press team within the next 48 hours. Your manuscript will remain under strict press embargo until 2 pm Eastern Time on the date of publication. For more information please contact onepress@plos.org.

If we can help with anything else, please email us at plosone@plos.org. 

Thank you for submitting your work to PLOS ONE and supporting open access. 

Kind regards, 

PLOS ONE Editorial Office Staff

on behalf of

Dr. Martial L Ndeffo Mbah 

Academic Editor

PLOS ONE
